# Manganese reduction and associated microbial communities in Antarctic surface sediments

**DOI:** 10.3389/fmicb.2024.1398021

**Published:** 2024-07-03

**Authors:** Lea C. Wunder, Inga Breuer, Graciana Willis-Poratti, David A. Aromokeye, Susann Henkel, Tim Richter-Heitmann, Xiuran Yin, Michael W. Friedrich

**Affiliations:** ^1^Microbial Ecophysiology Group, Faculty of Biology/Chemistry, University of Bremen, Bremen, Germany; ^2^Instituto Antártico Argentino, San Martín, Buenos Aires, Argentina; ^3^Consejo Nacional de Investigaciones Científicas y Técnicas (CONICET), Buenos Aires, Argentina; ^4^Alfred Wegener Institute Helmholtz Centre for Polar and Marine Research, Bremerhaven, Germany; ^5^State Key Laboratory of Marine Resource Utilization in South China Sea, Hainan University, Haikou, China; ^6^MARUM – Center for Marine Environmental Sciences, University of Bremen, Bremen, Germany

**Keywords:** manganese reduction, Potter Cove, marine surface sediment, *Desulfuromusa*, Sva1033, *Arcobacteraceae*, Antarctic, organic carbon degradation

## Abstract

The polar regions are the fastest warming places on earth. Accelerated glacial melting causes increased supply of nutrients such as metal oxides (i.e., iron and manganese oxides) into the surrounding environment, such as the marine sediments of Potter Cove, King George Island/Isla 25 de Mayo (West Antarctic Peninsula). Microbial manganese oxide reduction and the associated microbial communities are poorly understood in Antarctic sediments. Here, we investigated this process by geochemical measurements of *in situ* sediment pore water and by slurry incubation experiments which were accompanied by 16S rRNA sequencing. Members of the genus *Desulfuromusa* were the main responder to manganese oxide and acetate amendment in the incubations. Other organisms identified in relation to manganese and/or acetate utilization included *Desulfuromonas*, Sva1033 (family of *Desulfuromonadales*) and unclassified *Arcobacteraceae*. Our data show that distinct members of *Desulfuromonadales* are most active in organotrophic manganese reduction, thus providing strong evidence of their relevance in manganese reduction in permanently cold Antarctic sediments.

## 1 Introduction

Organic matter degradation in marine sediments is an important part of the global carbon cycle. The flux of carbon in marine sediments is regulated by microbial mineralization of organic matter to CO_2_ (LaRowe et al., 2020 and references therein) which contributes as potent greenhouse gas to global warming and drives the climate change (Mitchell, 1989). In anoxic sediments, the degradation of organic matter is performed by microorganisms in multiple steps. Briefly, polymeric carbon compounds are first hydrolyzed, then monomers are fermented and finally fermentation intermediates are oxidized coupled to the reduction of terminal electron acceptors such as nitrate, metal oxides and sulfate (Jørgensen, 2006).

Polar regions such as the Arctic and Antarctic are especially affected by climate change and are therefore warming-up multiple times faster than other areas of earth (Rantanen et al., 2022; Casado et al., 2023). Elevated temperatures result in accelerated melting of sea ice and glaciers and consequently increased meltwater input into the surrounding environment. Meltwater of sea ice and glaciers deliver nutrients, including different metals, into the ocean and underlying sediments (Death et al., 2014; Herbert et al., 2020; Forsch et al., 2021). There, the metal (iron and manganese) oxides can be used as terminal electron acceptors for microbial organic matter oxidation (Canfield et al., 1993b).

Microorganisms can couple manganese reduction to the oxidation of organic and inorganic electron donors (e.g., lactate, acetate, formate, hydrogen) (Burdige, 1993; Thamdrup, 2000). Reduced sulfur compounds such as elemental sulfur, thiosulfate or sulfide can also be used by microorganisms as electron donors for manganese reduction (Burdige, 1993; Thamdrup, 2000) and potentially contribute to a cryptic sulfur cycle (Aller and Rude, 1988). Bacteria associated with manganese reduction belong to different taxa such as *Pelobacter*, *Colwelliaceae, Arcobacteraceae, Shewanellaceae*, and *Oceanospirillaceae* (Thamdrup et al., 2000; Vandieken et al., 2012; Cho et al., 2020) and many iron reducing bacteria are also capable of reducing manganese oxides (Lovley, 1991; Vandieken et al., 2006a).

Microbial manganese reduction contributes up to 45% to carbon mineralization in manganese oxide rich sediments (Canfield et al., 1993a; Hyun et al., 2017). This process can also play a role in extreme environments such as the polar regions. It has been studied in different Arctic, manganese oxide rich sediments of the Barents Sea (Vandieken et al., 2006c), Beaufort Sea (Magen et al., 2011) and Svalbard fjords (Vandieken et al., 2006a; Wehrmann et al., 2014). There are only few studies that investigated specific organic matter degradation processes, e.g., iron reduction, and the related microbial communities in Antarctic subsurface sediments (Monien et al., 2014a; Aromokeye et al., 2021; Wunder et al., 2021; Baloza et al., 2022, 2023; Burdige and Christensen, 2022). The occurrence of manganese reduction and the respective microbial players have not been studied in any Antarctic sediments yet.

Our study site Potter Cove (King George Island/Isla 25 de Mayo, West Antarctic Peninsula) is heavily influenced by a glacier, which retreats rapidly turning from sea- to mostly land-terminating in the last decades (>1 km between 1956 and 2008; Rückamp et al., 2011; Neder et al., 2022). In the fjord sediments, iron oxides have been shown to be prominent terminal electron acceptors of organic matter degradation based on pore water iron concentrations (Monien et al., 2014a; Henkel et al., 2018) and incubation experiments (Aromokeye et al., 2021). The microbial processes using other possible electron acceptors for anaerobic organic matter degradation processes have not been studied yet at this site, but are potentially occurring due to the presence of dissolved manganese in the pore water, sufficient manganese oxide content (over 0.15 wt.%; Monien et al., 2014b) in the sediment and an abundance of sulfate (in average 20 mM across the cove; Monien et al., 2014a; Henkel et al., 2018).

Here, we explore manganese oxide as electron acceptor for organic matter degradation in Potter Cove sediments, Antarctica. We use geochemical profiles to indicate the likelihood of ongoing *in situ* manganese reduction, and further investigate the prerequisites of the process in terms of electron donor with sediment slurry incubations at an environmentally relevant temperature of 2°C. Finally, we identified *Desulfuromusa* as the active microbial player for manganese reduction by amplicon sequencing.

## 2 Materials and methods

### 2.1 Sampling site and *in situ* data

**Figure 1:**
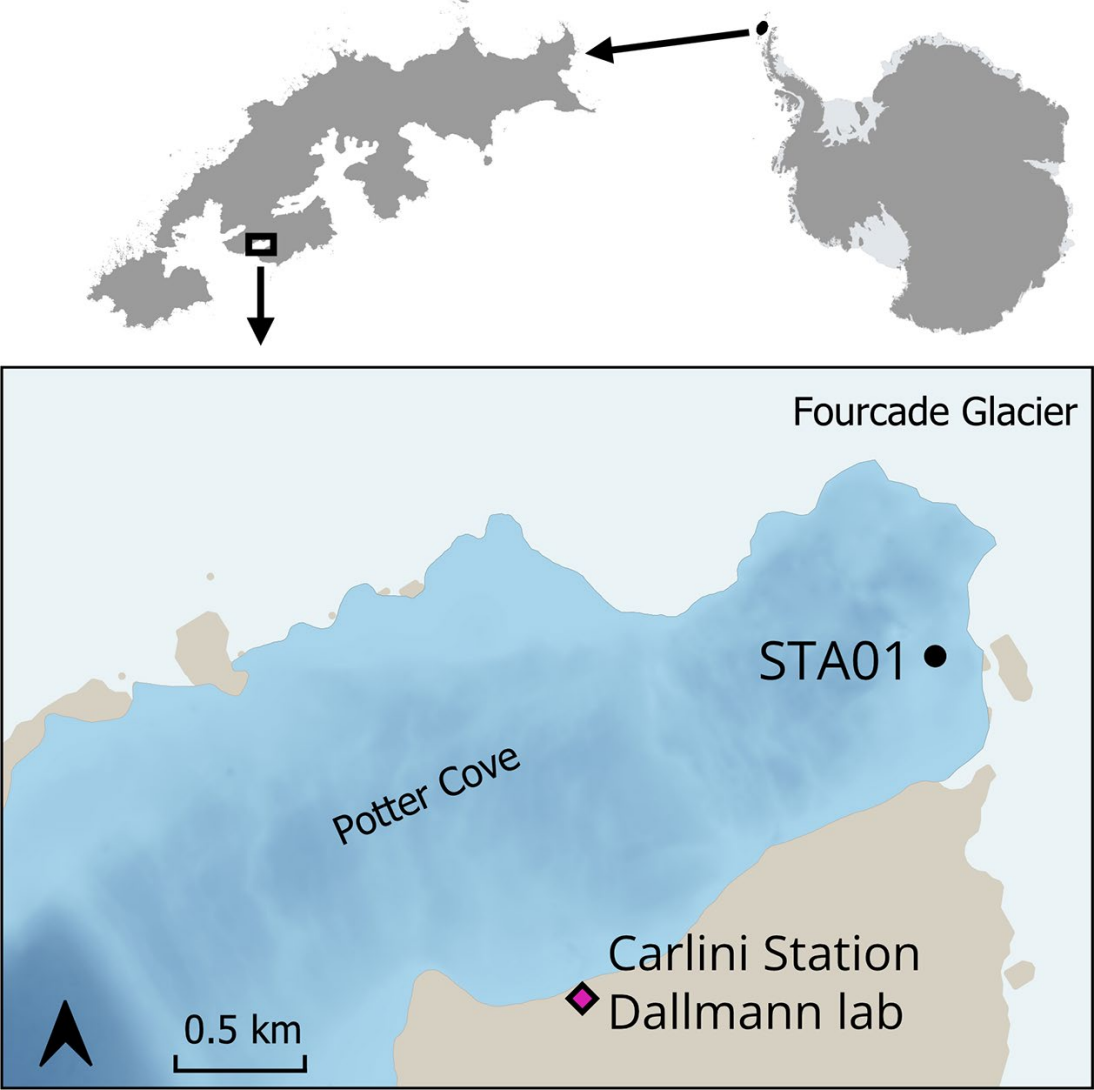
Sediment cores were taken at STA 01 in Potter Cove, King George Island/Isla 25 de Mayo, in close proximity to the Fourcade glacier. Map created with QGIS 3.34.3, bathymetry data (Neder et al., 2022) updated from Jerosch et al. (2015), basemap data from SCAR Antarctic Digital Database 2023, rock outcrop from Gerrish (2020) manually smoothed.

Sediment samples were collected in Potter Cove during austral summer 2018/2019. Sampling sites were selected based on previous geochemical measurements (Monien et al., 2014a; Henkel et al., 2018). For this study, sediments from Station 01 (STA 01) were used for further slurry preparation. STA 01 is located in close proximity of the glacial front (Figure 1, S62°13′34.16″/W58°38′25.1″). Five sediment replicate cores (25–35 cm length, diameter 60 mm) were retrieved with a hand-held gravity corer (USC 06000, UWITEC, Austria) and carefully transported in a vertical position to the Dallmann Laboratory at the Argentine Research Carlini Station. Two replicate cores were used for pore water collection with Rhizons (Rhizosphere Research Products, Netherlands) (Seeberg-Elverfeldt et al., 2005) in 1-cm intervals for the top 10 cm, followed by 2-cm intervals for the rest of the core. An aliquot of pore water samples (400 μL) was fixed with 100 μL of 0.5 M HCl for dissolved Fe^2+^ measurements, which was immediately determined in the Dallmann Laboratory. Another aliquot of the pore water was fixed with 50 μL 35% ultra-pure HCl and used for dissolved manganese and other cations determination at the Alfred Wegener Institute Helmholtz Centre for Polar and Marine Research (AWI). Pore water samples for sulfate measurement were not fixed with Zn-acetate as no sulfide smell was detected during sampling.

The other three replicate cores were used for solid phase sampling, and sediment samples were collected in the same depth resolution as for pore water. At each depth, part of the sediment was frozen at −20°C for analyzing the *in situ* microbial community by Illumina 16S rRNA gene amplicon sequencing. The rest of the sediment core was pooled in 5-cm depth-sections and stored at 2°C in 250-ml Schott-bottles sealed with rubber stoppers under N_2_ headspace (99.999% purity, Linde, Germany). The sediments were kept at 2°C in the dark for 2 years until the start of incubation experiments in the home laboratory.

### 2.2 Slurry incubations

In order to investigate the potential for manganese reduction in the Antarctic sediments collected and the microorganisms involved, slurry incubation experiments were conducted. Stored sediment from core STA01-02 was used for slurry incubations, pooling the whole length of the core (25 cm). The slurry was prepared in 1:5 ratio with sulfate-free artificial sea-water [26.4 g NaCl, 11.2 g MgCl_2_ · 6 H_2_O, 1.5 g CaCl_2_ · 2 H_2_O, 0.7 g KCl per liter; prepared with purified water (Milli-Q)] under flushing with N_2_ (99.999% purity, Linde, Germany). Slurry (30 mL) was distributed into 60-ml serum bottles and sealed with butyl rubber stoppers under N_2_ headspace. After a pre-incubation of 5 days at 2°C in the dark, substrates were added. Six different treatments were prepared in triplicates; four treatments contained a final concentration of 10 mM of the manganese oxide birnessite (MnO_2_, synthesized after McKenzie, 1971) as electron acceptor and (1) elemental sulfur (solid, crystalline orthorhombic sulfur S_8_, 1 mmol per liter added), (2) thiosulfate (S_2_O_3_^2−^, 1 mM, sodium salt), (3) acetate (1 mM, sodium salt), or (4) nothing as electron donor. All treatments without acetate contained 20 mM dissolved inorganic carbon (DIC, NaHCO_3_) as carbon source. Two control treatments with (5) 1 mM acetate or (6) 20 mM DIC were prepared. A treatment containing only birnessite and DIC and an additional DIC-only control treatment were set up in spring 2023. All the other incubations were performed in summer 2021, but were otherwise conducted the same way (see Supplementary material for more details).

The incubations ran for 20 days and were sampled over time for geochemical measurements and nucleic acid extraction. At each sampling time point, 2 mL of slurry was taken anoxically using a nitrogen-flushed syringe, centrifuged (20817 *g*, 4°C, 10 min) in a nitrogen pre-flushed 2-ml tube and the supernatant was fixed according to the parameter of interest. For dissolved manganese measurements, 0.5 mL supernatant was fixed with 50 μL 35% ultra-pure HCl and stored in the dark at 4°C until the measurement. For dissolved Fe^2+^ measurements, 100 μL of the supernatant was directly injected into the ferrozine reagent (see next section). For sulfide measurements, the supernatant was fixed as ZnS with a final concentration of 1.5% Zn-acetate and measured at the same day. For treatments containing thiosulfate, the ZnS pellet was washed with Milli-Q water and resuspend in the same volume of Milli-Q water to prevent interference of thiosulfate with the measurement reagents. For sulfate measurements, supernatant samples were fixed with a final concentration of 0.5% Zn-acetate to prevent the oxidation of free sulfide and samples were stored in the dark at 4°C until measurement. Samples for nucleic acid extraction were frozen at −80°C until further processing.

### 2.3 Geochemical methods

Dissolved manganese was measured by inductively coupled plasma optical emission spectrometry (ICP-OES) analysis (iCAP7400, Thermo Fisher Scientific Inc.) using an internal yttrium standard to correct for differences in the ionic strength of samples. The limit of quantification of the method was 1.35 μM Mn. No distinction between redox states of manganese is possible with this method. In all samples dissolved Fe^2+^ was measured following the ferrozine method (Viollier et al., 2000). In incubation samples sulfide (H_2_S, HS^−^, S^2−^) was measured following the methylene-blue method by Cline (1969). Absorbance measurements for Fe^2+^ and sulfide determination were performed on a spectrophotometer (Libra S12, Biochrom, Berlin, Germany). Sulfate was measured by ion chromatography equipped with a conductivity detector (incubation samples Metrohm 930 Compact IC Flex; *in situ* samples Metrohm Compact IC 761, Metrohm, Filderstadt, Germany).

### 2.4 Nucleic acid extraction and sequencing

In order to explore the bacterial community *in situ* and during the incubation, nucleic acids were extracted from 0.5 g frozen *in situ* sediment in depth intervals described above, and of 4 mL slurry of the incubation experiment samples. Combined extraction of RNA and DNA was performed using a phenol-chloroform protocol modified from Lueders et al. (2004) as follows: during precipitation with polyethylene glycol 6000, the samples were incubated at 4°C for 30 min and centrifuged at 20817 *g* at 4°C for 45 min. Nucleic acid pellets were washed twice with ice-cold 70% ethanol and eluted in 50 μL diethyl pyrocarbonate (DEPC) treated water (detailed protocol in Supplementary material). Nucleic acid extract quality was evaluated spectrophotometrically with a NanoDrop 1000 (Peqlab Biotechnologie, Erlangen, Germany) using A260/A230 and A260/A280 ratios (Olson and Morrow, 2012). For slurry nucleic acid extracts, DNase treatment was performed on subsamples (DNA-*free*^™^ Kit, Thermo Fisher Scientific) and cDNA was synthesized (GoScript^™^ Reverse Transcriptase Kit, Promega) for 16S rRNA sequencing following manufacturers’ instructions. DNA and cDNA concentrations were quantified with PicoGreen (Quant-iT PicoGreen^™^ dsDNA Assay Kit, Invitrogen^™^, Thermo Fisher Scientific) measured on a Fluoroskan Ascent FL fluorometer (Thermo Fisher Scientific).

PCR reactions for DNA of *in situ* samples and DNA and cDNA of slurry samples were performed targeting the bacterial 16S rRNA gene V4 region with the primer pair Bac515F (5′-GTGYCAGCMGCCGCGGTAA-3′) (Parada et al., 2016) and Bac805R (5′-GACTACHVGGGTATCTAATCC-3′) (Herlemann et al., 2011) in preparation of Illumina sequencing. Each primer had a unique 8-bp barcode at the 5′-end attached (Hamady et al., 2008), which allowed multiplexing of several samples in the same sequencing library. The PCR reaction (50 μL) contained 1x KAPA HiFi buffer, 0.3 mM dNTP mix, 0.02 U KAPA HiFi DNA polymerase (KAPA Biosystems), 0.2 mg/mL bovine serum albumin (BSA), 1 mM MgCl_2_, 1.5 μM each of forward and reverse primer and 2 ng template. The thermal cycling program was initial denaturation at 95°C for 5 min, 28 cycles of denaturation 98°C: 13 s, annealing 60°C: 20 s, elongation 72°C: 20 s followed by final elongation at 72°C for 1 min. PCR products were checked by agarose gel electrophoresis. If unspecific bands were visible, the PCR for the sample was repeated with reduced template amount. PCR products were purified (Monarch PCR and DNA Cleanup Kit, New England Biolabs, Germany) and quantified with PicoGreen^™^. PCR products were pooled in equimolar amounts per sequencing library. Further PCR-free sequencing preparations, including ligation of sequencing adapters, and paired-end 2× 250 bp Illumina sequencing (Novaseq6000 platform) was performed by Novogene Co. Ltd. (Cambridge, UK).

### 2.5 Sequence analysis

Sequencing data were analyzed following Hassenrueck (2022) based on the sequencing pipeline as described in Callahan et al. (2016). Demultiplexing and primer clipping was done with cutadapt (version 3.1, Martin, 2011). All following steps were performed in the R software (version 4.3.1, R Core Team, 2023) using the dada2 R package (version 1.28.0, Callahan et al., 2016). Forward (R1) and reverse reads (R2) were filtered to a maximum number of 2 expected errors. They were truncated to a summed length of 290 bp (R1 and R2) according to their quality profiles individually per sequence lane (Supplementary Table S1 for details). Error learning and correction was performed using a modified version of the loessErrfun function (Salazar, 2021) until convergence was reached. Reads were dereplicated and denoised into amplicon sequence variants (ASVs) and paired forward and reverse reads were merged. Reverse-complement sequences were turned around and chimeras were removed. Sequences outside of a range of 249–254 bp were discarded with the exception of 276 and 300 bp long sequences (details for sequence length selection see Supplementary material). These long ASVs were recovered from previously unmerged reads and mapped against the SILVA database (SSU Ref NR 99 release 138.1, Quast et al., 2012) using bbmap (version 38.86, Bushnell, 2014) and were merged accordingly. Taxonomic assignment was done with the SILVA database (SSU Ref NR 99 release 138.1, Quast et al., 2012) using a bootstrap value of 80.

For subsequent steps, the R packages taxa (version 0.4.2, Foster et al., 2018), metacoder (version 0.3.6, Foster et al., 2017), phyloseq (version 1.42.0, McMurdie and Holmes, 2013) and tidyverse (version 2.0.0, Wickham et al., 2019) were used. ASVs associated outside bacteria or to mitochondria or chloroplasts were removed and subsequently singletons and doubletons were removed. Reads per sample were normalized by relative abundance calculation and sufficient sequencing depth was checked with rarefaction curves (iNEXT package version 3.0.0, Chao et al., 2014; Supplementary Figures S1, S2).

For the taxa *Arcobacteraceae*, *Desulfuromusa*, *Desulfuromonas* and Sva1033 the most abundant ASVs in the incubation experiment were selected and the blastn tool was used to find the most similar sequences in the NCBI database (blastn version 2.14.1+, Altschul et al., 1997, Supplementary Tables S2–S5; for detailed settings see Supplementary material). A dissimilarity matrix was calculated for ASV sequences of *Desulfuromusa* from this study and a previous study of Potter Cove (Aromokeye et al., 2021) and 16S rRNA gene sequences of *Desulfuromusa* type strains using megablast (Zhang et al., 2000; Supplementary Table S6, for more details and accession no. see Supplementary material).

### 2.6 Statistical analysis of geochemical data

The general linear hypothesis test (Herberich et al., 2010) was used to distinguish at which time points the dissolved manganese concentrations differed significantly (*p* < 0.05) between treatments. For each individual time point, a linear model was created using the “lm” function with default settings and multiple comparisons between treatments were done using the function “glht” with the “Tukey” setting in the R package multcomp (version 1.4.25, Hothorn et al., 2008). Note, that this procedure does not require any assumption regarding the distribution of data points, sample sizes or variance homogeneity.

## 3 Results

### 3.1 Geochemistry of *in situ* sediments reveals potential for manganese reduction

**Figure 2:**
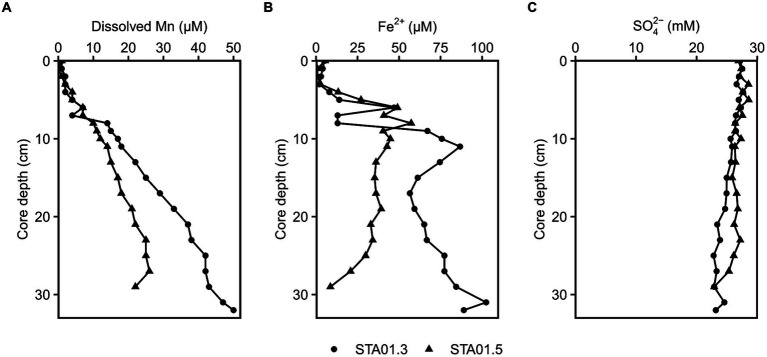
Geochemical parameters in the pore water of duplicate cores from STA 01 over sediment depths. **(A)** Dissolved manganese. **(B)** Dissolved Fe^2+^. **(C)** Sulfate. Duplicate cores STA01-3 (●) and STA01-5 (▲) of the same station are distinguished as indicated.

Different geochemical parameters (dissolved manganese, ferrous iron and sulfate) were measured in pore water of duplicate sediment cores of STA 01 to identify the dominant terminal electron accepting processes (Figure 2). Dissolved manganese concentrations increased linearly with depth up to 20 or 50 μM at the end of the core, while ferrous iron concentrations peaked with 55–85 μM at around 10 cm core depth. The presence of dissolved manganese and ferrous iron along the depth indicates ongoing manganese and iron reduction in the studied sediments. Sulfate concentrations decreased with core depth from 27 mM down to 23 mM. Hydrogen sulfide as proxy for sulfate reduction was undetected throughout both cores.

### 3.2 Manganese reduction observed in incubation experiments

**Figure 3:**
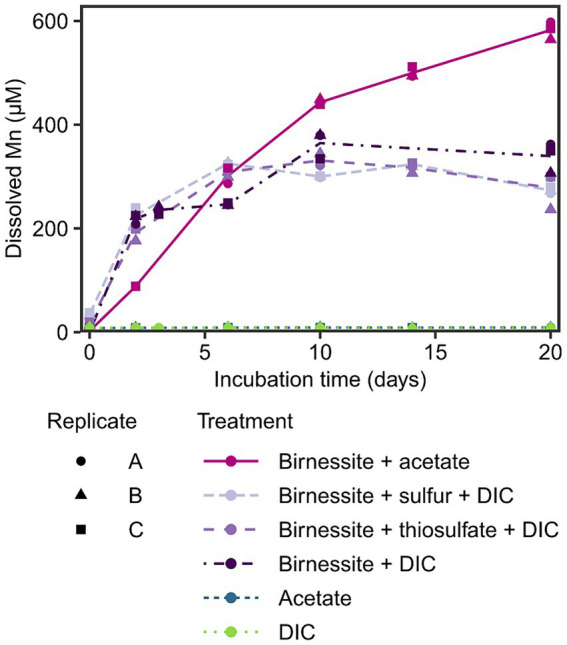
Dissolved manganese concentration in the incubation experiment over time. Different treatments are displayed by color and line type, and incubation triplicates by shape. The lines connect calculated means per treatment. Note, acetate and DIC treatment data points overlap.

To investigate the potential for manganese reduction in the sediments, anoxic slurry incubations were conducted using the manganese oxide birnessite as electron acceptor and acetate, elemental sulfur or thiosulfate as electron donors at 2°C (*in situ* temperature) for 20 days. An increase of dissolved manganese (Mn) up to 300–600 μM was detected in all treatments containing birnessite, while in control treatments without manganese oxide, the dissolved Mn remained below 10 μM (Figure 3). The effect of amended electron donors on the dissolved Mn concentration was only significant (*p* < 0.05) when acetate and birnessite were added (Supplementary Figure S3), resulting in the highest dissolved Mn concentration of 600 μM after 20 days. The addition of elemental sulfur or thiosulfate and birnessite resulted in similar dissolved Mn concentrations of up to 350 μM compared to the control treatment with only birnessite + DIC.

Sulfide, sulfate and dissolved ferrous iron were also monitored during incubation time. Ferrous iron remained <8 μM and sulfide <45 μM during the incubation time (Supplementary Figure S4). No additional sulfate was provided, so it ranged from 0.5–2 mM, decreasing between day 10 and 20 in treatments birnessite + acetate, acetate-only and DIC-only (Supplementary Figure S4).

### 3.3 Identification of microbial manganese reducers

**Figure 4:**
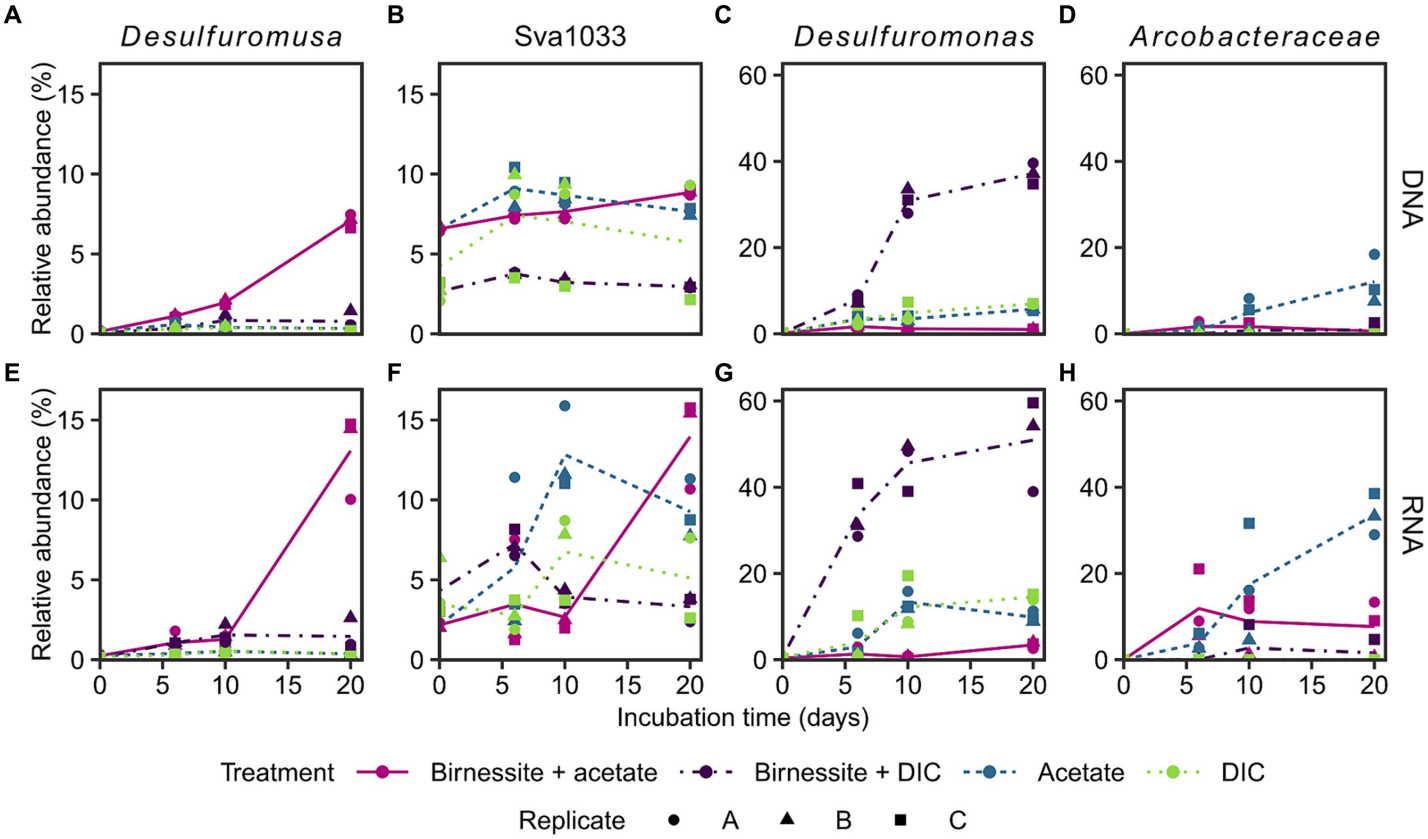
Relative abundance of 16S rRNA gene (**A–D**: DNA) and 16S rRNA (**E–H**: RNA) of most stimulated bacterial taxa (see text) in incubation experiment. Different treatments are displayed by color and line type, and incubation triplicates by shape. The lines connect calculated means per treatment. Sva1033 is a family of *Desulfuromonadales*. Plotted *Arcobacteraceae* were unclassified on genus level. Treatment birnessite + DIC was conducted separately and DIC replicate C is the associated control (see Supplementary material). For time point day 0, the associated slurry sample is plotted for all treatments (Supplementary Figures S5, S6). Note the different y-scales.

The active bacterial community was identified by 16S rRNA sequencing (RNA), supplemented with the complete bacterial community by 16S rRNA gene sequencing (DNA). Presumably, tracking 16S rRNA enables detecting potentially active microorganisms, whereas an increase in 16S rRNA gene abundance might enable following microbial growth (Blazewicz et al., 2013; Foesel et al., 2014). Only communities of the treatments birnessite + acetate and the associated controls, i.e., acetate-only, birnessite + DIC, DIC-only, were sequenced, as only here the electron donor coupled to manganese reduction could be identified (Figure 3). The stimulated microorganisms detected were unclassified members of the *Arcobacteraceae* family (class *Campylobacteria*) and members of the *Desulfuromonadales* order (class *Desulfuromonadia*), i.e., *Desulfuromusa*, *Desulfuromonas* and Sva1033 (uncultured family) (Figure 4). Other microorganisms that were not clearly stimulated by substrate addition, i.e., detected by 16S rRNA and 16S rRNA gene sequencing, but not increasing up to day 20, were members of *Desulfobacterales*, *Desulfobulbales*, *Gammaproteobacteria* and other less abundant groups <10% relative abundance (Supplementary Figures S5, S6).

*Desulfuromusa* was the only taxon stimulated by the addition of both, acetate and birnessite and not by addition of acetate or birnessite alone (Figures 4A,E). In the birnessite + acetate treatment it reached relative abundances of up to 15 and 7% for 16S rRNA and 16S rRNA gene, respectively, after 20 days, while in all controls the relative abundance remained <0.5% (<3% for birnessite + DIC) for both 16S rRNA and 16S rRNA gene. The peak in relative abundance of *Desulfuromusa* concurred with the maximum concentration of dissolved manganese after 20 days (Figure 3). The *Desulfuromusa* ASV had the closest BLAST hit with the uncultured *Desulfuromusa* sp. Fe30-7C-S previously identified in Black Smoker Chimneys (Caldera Vent Field, Takai et al., 2009; 100% 16S rRNA gene sequence identity, Supplementary Table S4). The closest cultured relative was *Desulfuromusa bakii* (99.6% 16S rRNA gene sequence identity, Supplementary Tables S4, S6).

The taxon Sva1033 was already present with 6% relative 16S rRNA gene abundance (DNA level) at the start of the incubation and only increased slightly (max. 9% at day 20 in birnessite + acetate, Figure 4B) in all sequenced treatments over time. However, Sva1033 16S rRNA (RNA level) increased from 3% to over 10% relative abundance in treatments birnessite + acetate and the associated acetate-only control in 20 days with a more rapid reaction in the acetate-only treatment (Figure 4F).

*Arcobacteraceae* was only stimulated by the addition of acetate with 16S rRNA gene (DNA level) abundance increasing from <0.2% up to 18% at day 20 (Figure 4D) and more rapid increase of 16S rRNA (RNA level) abundance to already 10% at day 6, all the way to >40% at day 20 (Figure 4H). In the control treatments without acetate addition, the 16S rRNA abundance remained <1%, except for one higher birnessite + DIC replicate C (Figures 4D,H). *Desulfuromonas*, as the only microbial player, showed a clear response in the birnessite + DIC treatment increasing rapidly in 16S rRNA and 16S rRNA gene abundance from 0.5% at day 0 to up to 60 and 40%, respectively, on day 20 (Figures 4C,G). The response in the other control treatments with DIC-only and acetate-only was much lower (max. 15% 16S rRNA day 10, Figure 4G) and reached only 4% in the birnessite + acetate treatment at day 20 with an even lower response on 16S rRNA gene level (2–7% day 20, Figure 4C).

In control treatments containing DIC, *Sulfurimonas* (class *Campylobacteria*) increased in 16S rRNA and 16S rRNA gene abundance up to 33 and 15%, respectively by day 20, but were < 3% at earlier timepoints (Supplementary Figures S5, S6). Some members of *Desulfocapsaceae* peaked on relative 16S rRNA abundance at day 10, but then decreased again at day 20, corroborated by no detectable increase of relative 16S rRNA gene abundance (Supplementary Figure S7).

The taxa enriched in the incubation experiment were also present in the *in situ* sediments (Supplementary Figure S8). However, only Sva1033 (class *Desulfuromonadia*) was present with high relative abundances of 16S rRNA genes up to 10%, while *Desulfuromusa* (class *Desulfuromonadia*) reached only 0.2% relative abundance and *Desulfuromonas* (class *Desulfuromonadia*) and *Arcobacteraceae* (class *Campylobacteria*) were < 0.1% relative abundance.

## 4 Discussion

Anaerobic organic carbon mineralization to CO_2_ can be coupled to different electron acceptors such as manganese oxides, iron oxides or sulfate (Jørgensen, 2006). The contribution of manganese reduction is often overlooked and thought to be confined to very shallow sediment depths where manganese oxides deplete in the top 2 cm (Thamdrup, 2000) or extremely manganese oxide rich sediments (e.g., Ulleung Basin 200 μmol/cm^3^, Hyun et al., 2017). Thamdrup (2000) proposed microbial manganese reduction only as important process if the manganese oxide concentration in the sediment is >20 μmol/cm^3^ and penetrates deeper than 2 cm below the surface. At the study site Potter Cove, solid phase manganese oxides reach 20 μmol/cm^3^ at the bottom of the 20 cm long core (recalculated from wt.%, Monien et al., 2014b; Supplementary material; Supplementary Figure S9). However, the availability of the electron acceptor alone does not warrant its reduction. For this, dissolved Mn in the pore water serves as indicator (Canfield et al., 1993a). At the sampling station STA 01, dissolved Mn accumulated with depth up to 50 μM (Figure 2). Pore water profiles of sediments taken 7 years earlier (2011) even showed accumulations up to 200 μM closer to the glacier front (station P04 in Monien et al., 2014a) and up to 100 μM in close proximity to STA 01 of this study (station P01 in Monien et al., 2014a, data replotted Supplementary Figure S9 from Monien et al., 2014b). The concentration differences might be due to local variability in the sediment or due to change of the environment, e.g., glacier retreat. Between austral summer 2013 and 2019, the glacier completely retreated from the shore closest to STA 01 (personal communication G. Willis-Poratti, M. sierra, Supplementary Figure S10) which might have had a direct influence on the sediment environment. Until now, there are no studies investigating manganese reduction in the Antarctic in detail. However, the process has been proposed to be relevant in a variety of Arctic sites, where dissolved Mn concentrations of a similar range were detected. For example, in sediments of Kongsfjorden and Van Keulenfjorden of Svalbard dissolved Mn reached 30–200 μM, and fluxes of 80 μmol m^−2^ d^−1^ Mn were calculated at stations with highest Mn concentrations (Wehrmann et al., 2014). In the Barents Sea and Baffin Bay, dissolved Mn accumulated up to 75–150 μM and 50 μM in the top 20 cm, respectively, and dissimilatory iron and manganese reduction were identified as important processes (Vandieken et al., 2006c; Algora et al., 2013, 2015). For Potter Cove, Monien et al. (2014a) suggested ongoing microbial manganese reduction based on the pore water profiles in the sediment, but the associated microbial community remained unknown. In this study, we aim to close this knowledge gap and provide first data for the Antarctic environment.

### 4.1 Manganese reduction also contributes to organic matter mineralization in Potter Cove sediments

After detecting manganese reduction in sediments of Potter Cove based on pore water profiles, the potential for active organotrophic manganese reduction was further confirmed by incubation experiments; dissolved Mn accumulation (up to 600 μM in 20 days) was observed when fresh manganese oxide (birnessite) was fed to slurry incubations with acetate as electron donor (Figure 3). These concentrations were similar to other manganese reducing incubation experiments with coastal or shelf sediments from Sweden, Ulleung Basin, Barents Sea or Beaufort Sea, with accumulation of 400–800 μM dissolved manganese within 10–20 days (Vandieken et al., 2006c, 2012, 2014; Magen et al., 2011; Hyun et al., 2017). However, in contrast to these previous studies, we added birnessite to selectively stimulate manganese reduction against other competing processes such as iron or sulfate reduction. The addition of the electron donor (i.e., acetate) alone was not sufficient to stimulate this process, however an accumulation of dissolved Mn was observed in the control incubation birnessite + DIC (Figure 3). This might have been caused by biotic or abiotic manganese reduction with internally produced or residual electron donors in the sediment. For example, manganese oxides can be reduced abiotically by the oxidation of residual FeS or pyrite (Schippers and Jørgensen, 2001), which is one scenario how the iron, manganese and sulfur cycle interplay with each other. However, no concurrent increase of the oxidation product sulfate was observed during the incubation period (Supplementary Figure S4). The addition of reduced sulfur compounds (elemental sulfur or thiosulfate) to the incubated sediments, as electron donors for probing lithotrophic manganese reduction, did not result in a statistically relevant distinction of dissolved Mn formation compared to the control incubations (Figure 3; Supplementary Figure S3). Therefore, we conclude that the tested reduced sulfur compounds could not serve as electron donors for microbial manganese reduction in these sediments. Other inorganic electron acceptors such as hydrogen or sulfide would need to be tested to exclude lithotrophic manganese reduction in general. Most likely, endogenous organic matter had served as electron donor for manganese reduction in control incubations.

It must be noted that dissolved Mn accumulation does not represent the rate of manganese reduction, as Mn^2+^ can rapidly adsorb to manganese and iron oxide surfaces and organic compounds or precipitate as MnCO_3_ or MnS (Suess, 1979; Burdige, 1993; Thamdrup, 2000). We exclude a large contribution of abiotic reduction of manganese oxide by ferrous iron produced by iron reduction (Thamdrup, 2000) as no additional fresh iron oxides were added. Yet, residual iron oxides in the incubated sediments were not sufficient to stimulate iron reduction activities beyond the 5 μM of ferrous iron detected, including in incubations supplemented with acetate (Supplementary Figure S4). We conclude that manganese reduction occurs mainly organotrophically and is not coupled to oxidation of tested reduced sulfur compounds in these sediments.

### 4.2 Active microbial players in organotrophic manganese reduction in Potter Cove sediments

The responsible microorganisms for organotrophic manganese reduction were investigated by 16S rRNA and 16S rRNA gene sequencing in treatments with acetate amendment and compared to control treatments. Four distinct taxa of microorganisms (*Desulfuromusa*, *Desulfuromonas*, *Arcobacteraceae* and Sva1033) were stimulated in the incubation experiment; *Desulfuromusa* was only active and grew when both, acetate and birnessite were supplied. Sva1033 (family of *Desulfuromonadales*) and *Arcobacteraceae* were also stimulated in the control treatments by the addition of acetate in the absence of birnessite, while *Desulfuromonas* was mainly stimulated in the birnessite + DIC control (Figure 4).

In the treatment with acetate and birnessite, the signature of manganese reduction (Figure 3) correlated with the stimulation of *Desulfuromusa* (Figures 4A,E). This taxon was found previously in RNA stable isotope probing experiments with Potter Cove sediment (100% identical ASVs, Supplementary Table S6), and it could use acetate with the addition of iron oxides and an electron shuttle in form of anthraquinone-2,6-disulfonic acid (AQDS) (Aromokeye et al., 2021). However, in these experiments, other microorganisms such as Sva1033 were more successful, i.e., more abundant, in thriving with iron oxides. Furthermore, *Desulfuromusa* was not detected in iron reducing incubation experiments by 16S rRNA gene sequencing only (Aromokeye et al., 2021), because apparently it prefers manganese oxide as electron acceptor at near *in situ* temperature conditions. Possibly, the same explanation applies for the lack of activity of *Desulfuromusa* in our incubations with only acetate. Furthermore, *Desulfuromusa* relied on supply of fresh organic compounds, i.e., acetate, as further confirmed by its lack of activity in the birnessite + DIC treatment (Figure 4E). In these control treatments, the other active microorganisms probably competed more successfully for remaining compounds in the sediment. In contrast, when both manganese oxide and acetate were added to our incubations, *Desulfuromusa* increased 50x in 20 days, even on 16S rRNA gene level (Figure 4A).

Members of the *Desulfuromusa* genus are known for utilizing acetate as electron donor (Finster and Bak, 1993; Liesack and Finster, 1994; Vandieken et al., 2006b). However, the use of manganese oxide as electron acceptor was only demonstrated in *Desulfuromusa ferrireducens* (Vandieken et al., 2006b), while all other isolated strains are able to use iron but were not tested for manganese reduction (Finster and Bak, 1993; Lonergan et al., 1996). The closest cultured relative of the *Desulfuromusa* strain found in our study is *Desulfuromusa bakii* (99.6% sequence identity, Supplementary Tables S4, S6). *Desulfuromusa* species were previously also detected in Arctic (Vandieken et al., 2006a,c; Buongiorno et al., 2019; Magnuson et al., 2023) and Antarctic environments (Bowman et al., 2000; Taboada et al., 2020). However, their presence was only linked to a potential activity of iron reduction in few cases (Vandieken et al., 2006a). The *Desulfuromusa* strain found in our study apparently does not compete for iron reduction or other residual electron acceptors at environmentally relevant temperature, which is in contrast to previous studies at different locations (Vandieken et al., 2006a; An et al., 2020; Hamdan and Salam, 2020). Possibly, the low, close-to-*in situ* temperature of 2°C used in our study promoted the manganese reducing activity of this microorganism, avoiding competition with the other present acetate utilizers. We propose that *Desulfuromusa* occupied a niche of organotrophic manganese reduction in glacially influenced sediments, providing strong evidence for its relevance in microbial manganese reduction in Antarctic sediments.

While *Desulfuromusa* was the only microbe which clearly performed manganese reduction in our incubation experiments, other microbes were stimulated in the various other control treatments not optimized for manganese reduction. For example, *Arcobacteraceae* increased in relative 16S rRNA and 16S rRNA gene abundance, but strongly only in the presence of acetate, less with birnessite and acetate and was nearly absent without it (Figures 4D,H). This agrees with reports of anaerobic acetate oxidation by this taxon (Vandieken and Thamdrup, 2013); still it was previously shown that they can also use manganese as electron acceptor (Vandieken et al., 2012).

Next, *Desulfuromonas* showed a strong positive response to the amendment of birnessite alone, but surprisingly not in the birnessite + acetate treatment, while increasing in the DIC-only and acetate-only controls (Figures 4C,G). The ASV mainly stimulated in the treatments (sq1) was most closely related to *Desulfuromonas svalbardensis* (100% sequence identity, Supplementary Table S3), which is known to couple iron or manganese reduction to oxidation of fermentation products such as acetate (Vandieken et al., 2006b). In our study, *Desulfuromonas* was likely outcompeted by other microorganisms utilizing the amended acetate when birnessite was provided. However, when only birnessite (and DIC) were provided to the sediment background, *Desulfuromonas* possibly competed more successfully for a different residual electron donor.

In contrast to the other described stimulated taxa which showed low abundances at the start of the experiment, the largely unexplored family Sva1033 (*Desulfuromonadales*) was already present at day 0 and still increased during the incubation in 16S rRNA abundance in acetate amended and associated DIC-only control treatments (5x increase, Figure 4F). At the study site, this taxon likely plays an important role as indicated by its high relative abundance in the *in situ* sediments (Supplementary Figure S8) and its high presence already at the start and throughout the experiment. This is in agreement with previous studies investigating Antarctic sediments (Aromokeye et al., 2021; Baloza et al., 2023) and sub-Antarctic sediments around South Georgia (Wunder et al., 2021). Sva1033 was shown before to oxidize acetate and reduce iron oxides in sediments of Potter Cove (Aromokeye et al., 2021; Wunder et al., 2021), and also here it showed higher relative 16S rRNA abundance in acetate amended treatments. The late 7-fold relative abundance increase in 16S rRNA in the birnessite + acetate treatment at day 20 (Figure 4F) even hints at a potential to switch to manganese oxides as electron acceptor after a period of adaptation, e.g., from iron reduction (Wunder et al., 2021).

Species of *Desulforhopalus* and *Desulfocapsaceae* also responded in the birnessite + acetate treatment within 6–10 days (Supplementary Figure S7). However, as these responses were only transient and did not increase further despite ongoing manganese reduction after day 10 (Figure 3), it was unclear whether these bacteria reacted to the substrate addition.

The microbial community linked to manganese reduction in other marine environments was so far mostly associated with *Pelobacter*, *Colwelliaceae, Arcobacteraceae, Shewanellaceae*, *Oceanospirillaceae*, or *Burkholderiales* (Thamdrup et al., 2000; Vandieken et al., 2012; Algora et al., 2015; Cho et al., 2020). Except for *Arcobacteraceae*, none of these other taxa were present or were only extremely low abundant in the incubations and in the *in situ* sediments (<0.05%, *Colwellia* 0.2% at 2 cm *in situ*, *Burkholderiales* < 1% in incubation). However, most of the previously studied sites for manganese reduction and the associated microbial community are extremely manganese-rich, e.g., Ulleung Basin and Skagerrak with 200–600 μmol/cm^3^ (Vandieken et al., 2012) versus 20 μmol/cm^3^ for Potter Cove (Supplementary Figure S9). Another parameter differing at these sites is temperature: most of the previous study sites are in temperate environments. In Arctic sediments from the deep basin of Baffin Bay, one of the locations where Mn reduction was investigated in a cold environment (Algora et al., 2013), the dissolved manganese concentrations were in a comparable range to Potter Cove with of 20–60 μM. Here, manganese reduction was proposed to be an important part of anaerobic organic matter degradation (Algora et al., 2013), but the associated microbial community was different again, suggesting *Betaproteobacteria* as potential manganese and iron reducers (Algora et al., 2015). Both, the availability of manganese oxides and the colder temperature might be responsible factors for a different manganese reducing community in our Antarctic sediment samples with the above discussed *Desulfuromusa* as key microorganism. Further investigations are needed to unravel influencing factors on the manganese reducing community on a larger, global scale.

### 4.3 Relevance of manganese reduction in rapidly warming Antarctic sediments

At the study site Potter Cove, the *in situ* geochemistry and the incubation experiments with manganese oxides clearly demonstrated the potential for the contribution of manganese reduction to organic matter degradation in the sediments. At other sampling locations in Potter Cove, elevated dissolved manganese concentrations up to even 200 μM indicate that this process might contribute to organic matter degradation there as well (e.g., stations P05, K48, in Figure 3 Monien et al., 2014a). The high dissolved ferrous iron concentrations of 100–800 μM detected in the pore water in our study (Figure 2) and multiple previous studies (Monien et al., 2014a; Henkel et al., 2018) indicate contribution of microbial iron reduction to organic matter degradation (Aromokeye et al., 2021). Iron was also shown as important electron acceptor in glacially or sea ice influenced sediments in the Arctic, such as Svalbard (Vandieken et al., 2006a,c) or Greenland (Glud et al., 2000), and also at the Antarctic shelf (Baloza et al., 2022). Rate measurements of carbon oxidation as conducted for some Arctic sediment (Vandieken et al., 2006c) would be necessary to investigate the contribution of the different processes, i.e., iron, manganese and sulfate reduction, in Potter Cove sediments.

An open question remains how glacial input influences manganese reduction in the marine sediment. For Potter Cove, it has been clearly shown that suspended particulate matter transported by surficial glacial meltwater supplies iron and manganese into the cove (Monien et al., 2017). There are many studies showing high amounts of iron supplied to the ocean and underlying sediments by subglacial or surficial meltwater (Wehrmann et al., 2014; Monien et al., 2017; Raiswell et al., 2018; Forsch et al., 2021). The bioavailability of this iron varies depending on the source, and subsequent biogeochemical cycling plays an important role to make this supplied iron bioavailable to, e.g., fuel primary production (Henkel et al., 2018; Herbert et al., 2021; Laufer-Meiser et al., 2021). Perhaps, the same applies to manganese and this study has demonstrated that the manganese supply from glacial input will likely increase the likelihood for manganese reduction to contribute to organic matter degradation in Antarctic sediments.

## 5 Conclusion

We show that organotrophic manganese reduction has the potential to contribute to organic matter mineralization in the Antarctic sediments of Potter Cove (King George Island/Isla 25 de Mayo). We identified *Desulfuromusa* as a key organism for organotrophic manganese reduction and propose that it occupies a niche of manganese reduction at a low, environmentally relevant temperature. *Arcobacteraceae* also contributed to organic carbon mineralization (i.e., acetate oxidation); however, this group likely did not reduce manganese but thrived on unidentified residual electron acceptors. *Desulfuromonas* was stimulated, but the underlying processes are not clear yet. The largely uncharacterized group Sva1033 (family of *Desulfuromonadales*) also contributed to acetate oxidation with the potential to link it to manganese oxide reduction which would extend the known utilized electron acceptors for this microorganism. The manganese reducing bacterial community identified in Potter Cove differed from other previously studied locations. This raises the question whether the observed diversity is caused by geographical location effects, i.e., whether Antarctica hosts a different microbial community than, e.g., the Arctic, or by other factors such as temperature, glacial influence, or sediment composition. In the context of a rapidly changing environment due to global warming, it is of interest to gain a better understanding of the ecology in the current environment to be able to predict future changes. This study brings us one step further by showing manganese reduction might become more relevant for organic matter degradation in permanently cold marine environments.

## Data availability statement

The datasets presented in this study can be found in online repositories. The names of the repository/repositories and accession number(s) can be found at: https://www.ebi.ac.uk/ena, PRJEB72873; https://www.ebi.ac.uk/ena, PRJEB72882; https://www.pangaea.de/; https://doi.pangaea.de/10.1594/PANGAEA.941109; https://github.com/Microbial-Ecophysiology/Mn-red-PotterCove.

## Author contributions

LW: Conceptualization, Formal analysis, Investigation, Visualization, Writing – original draft. IB: Conceptualization, Formal analysis, Investigation, Writing – review & editing. GW-P: Conceptualization, Funding acquisition, Investigation, Writing – review & editing. DA: Conceptualization, Investigation, Writing – review & editing. SH: Investigation, Writing – review & editing. TR-H: Writing – review & editing. XY: Conceptualization, Writing – review & editing. MF: Funding acquisition, Project administration, Writing – review & editing.
