# Supplementary material for: Manganese reduction and associated microbial communities in Antarctic surface sediments
Source: Front Microbiol. 2024 Jul 3;15:1398021. doi: 10.3389/fmicb.2024.1398021 (PMC11252027; doi:10.3389/fmicb.2024.1398021)
Supplement: Supplementary file 1 [file Data_Sheet_1.pdf]

Supplementary Material

1 Supplementary Data

1.1 Incubation set-up

Slurry incubations were prepared as described in the main manuscript. In total six treatments differing in their substrate addition were set up as shown in Table 1.

The substrate birnessite (MnO_2) was synthesized following McKenzie (1971). Briefly, 2 mol of concentrated HCl was added dropwise to a boiling solution of 1 mol $KMnO_4$ (SigmaAldrich, Taufkirchen, Germany) in 2.5 ml of sterile deionized water, under vigorous stirring. The mixture was boiled for 10- 15 min. During cooling of the mixture brownish precipitates settled which were washed 5 times with de-ionized water to remove impurities before transfer into centrifuge tubes. Wet precipitates were centrifuged 3 times at 3,834 *g* for 10 min at room temperature with a Sorvall Evolution RC Centrifuge (Thermo Scientific, Germany). After each centrifugation step, the supernatant was decanted and pellet was rinsed with de-ionized water. MnO_2 was then dispersed in anoxic, de-ionized water and sterilized. The suspension was stored in 120 ml serum flask under N_2 atmosphere (N_2, 99.999%).

The substrate elemental sulfur was prepared as crystalline orthorhombic sulfur (S_8) by dissolving 10 g commercially available sulfur (Applichem, Darmstadt, Germany) in 30 ml carbon disulfide (CS_2). Traces of CS_2 were removed by vigorous stirring of the solution sulfur at 60°C overnight. The S_8 was then ground and dispersed in anoxic, sterile and de-ionized water by long and vigorous shaking. The suspension was stored in 120-ml serum flask under N_2 atmosphere (N_2, 99.999%).

The control treatment birnessite + DIC was performed in 2023 while the rest of the experiment was performed in 2021. The sediment for slurry preparation in 2021 and 2023 originated from the same Schott-bottles used for storage. Control treatments only containing slurry and DIC were done in 2021 and in 2023 in order to check background activities. During the course of incubation, subsamples were taken for geochemical measurements as described in the main manuscript. Treatments containing only DIC set up in 2021 and 2023 behaved similarly for ferrous iron, sulfide and manganese (Figure S 4). However, in both treatments set up in 2023 (DIC_2023, Birnessite + DIC) the sulfate concentration was consistently 1 mM lower compared to treatments set up in 2021. We hypothesize that during the additional 2 years of storage of the sediment used for setting

up the slurry, additional remaining sulfate was used up in the sediment by sulfate reduction. This hypothesis was supported by high relative abundance on RNA level of microorganisms known for sulfate reduction such as members of *Desulfocapsaceae* and *Desulfobacteraceae* (Jørgensen et al., 2019) in the incubation already at day 0 (Figure S 7). However, these taxa showed no further relative abundance **increase** of 16S rRNA or 16S rRNA gene up to day 20 when the dissolved Mn concentrations was highest (Figure S 7). Thus, they were likely not involved in the stimulated process of manganese reduction in the incubations but might have thrived on concurrent sulfate reduction or their RNA was still present from the activity during sediment storage. This hypothesis is further supported by an increase of *Desulforhopalus* (*Desulfocapsaceae*) in the initial slurry from 2023 compared to 2021 (Figure S5, S6). A possible explanation for the lack of development of free sulfide is abiotic reactions with the slurry environment. Sulfide rapidly reacts with ferrous iron, iron or manganese mineral surfaces or could also be re-oxidized (Jørgensen, 1977; Canfield et al., 1993; Michaud et al., 2020).

We did not see a similar effect in 2021 vs. 2023 treatments in dissolved manganese and ferrous iron concentration, likely because any fresh, easily microbially available manganese or iron oxides were already used up by summer 2021. Manganese reducing activity could only be stimulated by the addition of fresh manganese oxide in the form of birnessite (Figure S 3). Here, treatments from 2021 and 2023 behaved similarly if birnessite was added (every treatment with birnessite but without acetate) or not (DIC-only treatments) (Figure S 4). Therefore, we concluded that sediments still behaved similarly in terms of the investigated process of manganese reduction in 2023 compared to 2021. The lower sulfate concentration did not influence the experiment in a way that would alter the conclusion about manganese reduction being influenced by acetate as electron donor, but not thiosulfate or elemental sulfur.

Table 1: Detailed set-up of substrate addition of slurry incubation experiment. Final concentrations of substrates are given in brackets.

Substrate/ Treatment	δ-MnO_2 (10 mM)	S^0 (1 mM)	$S_2O_3^{2-}$ (1 mM)	Acetate (1 mM)	DIC (20 mM)	Date set-up	Replicates
**Birnessite + acetate**	x			x		03.06.21	A, B, C
**Birnessite + sulfur + DIC**	x	x			x	03.06.21	A, B, C
**Birnessite + thiosulfate + DIC**	x		x		x	03.06.21	A, B, C
**Acetate**	x			x		03.06.21	A, B, C
**DIC**					x	03.06.21	A, B
**Birnessite + DIC**	x				x	02.02.23	A, B, C
**DIC_2023**					x	02.02.23	C

1.2 Nucleic acid extraction protocol for combined DNA and RNA extraction

Combined nucleic acid extraction of DNA and RNA was performed following a modified protocol from Lueders et al. (2004). Zirconium beads (heat sterilized at 180°C for 4 h, ~0.7 g) for bead beating were filled in a sterile screw cap tube. Maximum 0.5 g beads for extraction were added. 750 µl sodium phosphate buffer (120 mM, pH 8, 112.87 mM Na_2HPO_4 and 7.12 mM NaH_2PO_4 in RNase-free water, autoclaved) and 250 µl TNS solution (500 mM Tris-HCl pH 8.0, 100 mM NaCl, 10 % SDS (w/v), adjust pH with HCl, in RNase free water, autoclaved) were added. Bead beating was performed twice for 45 s at 6.5 m/s. Samples were kept on ice between bead-beating steps and during all steps afterwards. Samples were centrifuged for 20 min at 20817 *g* at 4°C. All following centrifugation steps were performed under the same conditions with varying times. The supernatant was transferred into a new, sterile 2-ml tube and 1 volume Phenol/Chloroform/Isoamyl alcohol (25:24:1, pH 4.5) was added, gently mixed and centrifuged for 5 min. The supernatant was transferred into a new, sterile 2-ml tube and 1 volume Chloroform/Isoamyl alcohol (24:1) was added, gently mixed and centrifuged for 5 min. The supernatant was transferred into a new, sterile 2-ml tube. Roughly 2 volumes PEG (30% (w/v) polyethylene glycol 6000 in 1.6 M NaCl, in RNAse free water, autoclaved) were added up to a volume of 2 ml. Samples were incubated at 4°C for 30 min followed by centrifugation for 45 min. Liquid was carefully removed with a pipet and the remaining nucleic

acid pellet was washed twice by adding 500 µl ice-cold 70% ethanol, centrifugation for 5 min and the careful removal of liquid. Nucleic acid pellets were briefly dried by placing the tubes on a normal rack with open lids for a maximum of 5 min under a fume hood. 50 µl DEPC-treated water were used for elution. Samples were checked on NanoDrop.

1.3 Sequence data analysis – ASV length distribution and rescuing fragments which exceed maximum insert size

The pipeline used to analyze the amplicon sequence data has been described in the main text; here details for retrieving long ASVs were provided.

During merging of forward and reverse reads, the paired reads are required to overlap by at least 10 bp with 0 mismatches. Merging of forward and reverse reads typically expects an overlap of 30 bp to account for variable lengths of 16S rRNA genes in the sequenced region of different microorganisms. Unmerged sequences were mapped against a reference data base and if aligning properly, were merged with their corresponding paired read and potential gaps were filled with ‘N’s. After chimera removal, the ASV length distribution was inspected manually, checking for number of ASVs and reads of different lengths. Usually, a normal distribution of ASV counts and read counts over ASV lengths is expected, with a clear peak around the expected insert size. For the datasets here, 99% of the reads and ASVs were found between 249 and 254 bp ASV length. However, there was a substantial amount of reads and ASVs at 276 or 300 bp (see Table 1). As a quality control, ASV sequences, which were outside of the ASV length range determined by the normal distribution (here 249-254 bp), were exported as fastq file. The ~ 20 ASVs with the most read counts were compared to a public database using BLAST (see below). Most of the ASVs outside the range only had a very low percent identity hits (< 80%) and were considered to be most likely sequence artifacts, so that they were discarded. However, ASVs belonging to the peak at 276 or 300 bp length were often associated to typical sulfur oxidizing bacteria such as *Sulfurimonas* or *Arcobacter* (Han and Perner, 2015; Jurado et al., 2021). Therefore, in this sequencing pipeline it was decided to keep these long ASVs.

1.4 BLAST and dissimilatory matrix of ASVs

For taxa enriched in the incubation experiment, the ASV abundance tables were checked for the top 1-4 most abundant ASVs for the taxa *Arcobacteraceae*, *Desulfuromonas*, *Desulfuromusa* and Sva1033. The sequences were uploaded to the online NCBI BLAST tool (https://blast.ncbi.nlm.nih.gov/Blast.cgi?PROGRAM=blastn&PAGE_TYPE=BlastSearch&LINK_LOC=blasthome). The standard database "Nucleotide collection (nr/nt)" was selected, the search was optimized for somewhat similar sequences selecting the search algorithm blastn (Altschul et al., 1997) version 2.14.1+. The search was performed on the 27.11.2023. For Sva1033 ASVs, uncultured and environmental samples were excluded, for the other taxa nothing was excluded. The received results were combined manually in a table (Table S 2 - Table S 5) by selecting the best two results in terms of coverage and identity, and every isolated organism within the top 100 hits.

A dissimilarity matrix was calculated in order to compare the similarity of the *Desulfuromusa* ASV found in the incubations of this study with *in situ* sequences of Potter Cove, previous experiments from Potter Cove (Aromokeye et al., 2021) and *Desulfuromusa* type strains (Table S 6). A fasta file was created containing all sequences for the dissimilatory matrix. The file was uploaded to the NCBI BLAST tool both as query and subject sequence. The megablast algorithm was selected for alignment (Zhang et al., 2000).

1.4.1 ASV sequences used for BLAST and dissimilatory matrix

> sq10;size=126178;Mn-incubations;Arcobacteraceae;

TACGGAGGGTGCAAGCGTTACTCGGAATCACTGGGCGTAAAGAGAATGTAGGCGGG
TTAATAAGTCAGAAGTGAAATCCAATAGCTCAACTATTGAACTGCTTTTGAAACTGT
TAGCCTAGAATATGGGAGAGGTAGATGGAATTTCTGGTGTAGGGGTAAAATCCGTA
GAGATCAGAAGGAATACCGATTGCGAAGGCGATCTACTGGAACATTATTGACGCTG
AGATTCGAAAGCGTGGGGAGCAAACA

> sq23;size=56069;Mn-incubations;Arcobacteraceae;

TACGGAGGGTGCAAGCGTTACTCGGAATCACTGGGCGTAAAGAGAATGTAGGCGGG
TTAATAAGTCAGAAGTGAAATCCAATAGCTCAACTATTGAACTGCTTTTGAAACTGT
TAGCCTAGAATATGGGAGAGGTAGATGGAATTTCTGGTGTAGGGGTAAAATCCGTA
GATATCAGAAGGAATACCGATTGCGAAGGCGATCTACTGGAACATTATTGACGCTG
AGATTCGAAAGCGTGGGGAGCAAACA

> sq60;size=17637;Mn-incubations;Arcobacteraceae;

TACGGAGGGTGCAAGCGTTACTCGGAATCACTGGGCGTAAAGAGAATGTAGGCGGG
TAGATAAGTCAGAAGTGAAATCCAATAGCTCAACTATTGAACTGCTTTTGAAACTGT
TTACCTAGAATATGGGAGAGGTAGATGGAATTTCTGGTGTAGGGGTAAAATCCGTA
GAGATCAGAAGGAATACCGATTGCGAAGGCGATCTACTGGAACATTATTGACGCTG
AGATTCGAAAGCGTGGGGAGCAAACA

> sq4;size=337342;Mn-incubations;Desulfuromonas;

TACGGAGGGTGCAAGCGTTGTTCGGAATTATTGGGCGTAAAGCGCGTGTAGGCGGT
TTGTTAAGTCTGATGTGAAAGCCCCGGGCTCAACCTGGGAAGTGCATTGGAAACTGG
CAAACTTGAGTACGGGAGAGGGAAGTGGAATTTCGAGTGTAGGGGTGAAATCCGTA
GATATTCGAAGGAACACCAGTGGCGAAGGCGGCTTCCTGGACCGATACTGACGCTG
AGACGCGAAAGCGTGGGGAGCAAACA

> sq1;size=856795;Mn-incubations;Desulfuromonas;

TACGGAGGGTGCAAGCGTTGTTCGGAATTATTGGGCGTAAAGCGCGTGTAGGCGGT
TAGTTAAGTCTGATGTGAAAGCCCCGGGCTCAACCTGGGAAGTGCATTGGATACTGG
CAAACTTGAGTACGGGAGAGGGAAGTGGAATTTCGAGTGTAGGGGTGAAATCCGTA
GATATTCGAAGGAACACCAGTGGCGAAGGCGGCTTCCTGGACCGATACTGACGCTG
AGACGCGAAAGCGTGGGGAGCAAACA

> sq18;size=80773;Mn-incubations;Desulfuromonas;

TACGGAGGGTGCAAGCGTTGTTCGGAATTATTGGGCGTAAAGCGCGTGTAGGCGGT
TAGTTAAGTCTGATGTGAAAGCCCCGGGCTCAACCTGGGAAGTGCATTGGAAACTG
GCAAACTTGAGTACGGGAGAGGGAAGTGGAATTTCGAGTGTAGGGGTGAAATCCGT
AGATATTCGAAGGAACACCAGTGGCGAAGGCGGCTTCCTGGACCGATACTGACGCT
GAGACGCGAAAGCGTGGGGAGCAAACA

> sq19;size=80707;Mn-incubations;Desulfuromonas;

TACGGAGGGTGCAAGCGTTGTTCGGAATTATTGGGCGTAAAGCGCGTGTAGGCGGT
TCGTTAAGTCTGATGTGAAAGCCCCGGGCTCAACCTGGGAAGTGCATTGGATACTGG
CAAACTTGAGTACGGGAGAGGGAAGTGGAATTTCGAGTGTAGGGGTGAAATCCGTA
GATATTCGAAGGAACACCAGTGGCGAAGGCGGCTTCCTGGACCGATACTGACGCTG
AGACGCGAAAGCGTGGGGAGCAAACA

> sq22;size=60363;Mn-incubations;Desulfuromusa;

TACGGAGGGTGCAAGCGTTGTTCGGAATTATTGGGCGTAAAGAGCATGTAGGCGGA
CTATTAAGTCTGGTGTGAAAGCCCGGGGCTCAACCCCGGAAGTGCATTGGATACTGG
TAGTCTTGAGTATGGGAGAGGAAAGTGGAATTCCGAGTGTAGGAGTGAAATCCGTA
GATATTCGGAGGAACACCAGTGGCGAAGGCGGCTTTCTGGACCAATACTGACGCTG
AGATGCGAAAGCGTGGGGAGCGAACA

>sq402;size=15546;in-situ-PotterCove;Desulfuromusa;

TACGGAGGGTGCAAGCGTTGTTCGGAATTATTGGGCGTAAAGAGCATGTAGGCGGT
CTGTTAAGTCTGGTGTGAAAGCCCGGGGCTCAACCCCGGAAGTGCATTGGATACTGG
CAGACTTGAGTATGGGAGAGGAAAGCGGAATTCCGAGTGTAGGAGTGAAATCCGTA
GATATTCGGAGGAACACCAGTGGCGAAGGCGGCTTTCTGGACCAATACTGACGCTG
AGATGCGAAAGCGTGGGGAGCGAACA

>sq875;size=6918;in-situ-PotterCove;Desulfuromusa;

TACGGAGGGTGCAAGCGTTGTTCGGAATTATTGGGCGTAAAGAGCATGTAGGCGGA
CTATTAAGTCTGGTGTGAAAGCCCGGGGCTCAACCCCGGAAGTGCATTGGATACTGG
TAGTCTTGAGTATGGGAGAGGAAAGTGGAATTCCGAGTGTAGGAGTGAAATCCGTA
GATATTCGGAGGAACACCAGTGGCGAAGGCGGCTTTCTGGACCAATACTGACGCTG
AGATGCGAAAGCGTGGGGAGCGAACA

>OTU33687944532408;Macroalgae-PotterCove;Desulfuromusa;

CAGCAGCCGCGGTAATACGGAGGGTGCAAGCGTTGTTCGGAATTATTGGGCGTAAA
GAGCATGTAGGCGGACTATTAAGTCTGGTGTGAAAGCCCGGGGCTCAACCCCGGAA
GTGCATTGGATACTGGTAGTCTTGAGTATGGGAGAGGAAAGTGGAATTCCGAGTGT
AGGAGTGAAATCCGTAGATATTCGGAGGAACACCAGTGGCGAAGGCGGCTTTCTGG
ACCAATACTGACGCTG

>OTU76210975478505;Macroalgae-PotterCove;Desulfuromusa;

GGACTACGGGGGTATCTAATCCTGTTCGCTCCCCACGCTTTCGCATCTCAGCGTCAG
TATTGGTCCAGAAAGCCGCCTTCGCCACTGGTGTTCCTCCGAATATCTACGGATTTC
ACTCCTACACTCGGAATTCCACTTTCCTCTCCCATACTCAAGACTACCAGTATCCAAT
GCACTTCCGGGGTTGAGCCCCGGGCTTTCACACCAGACTTAATAGTCCGCCTACATG
CTCTTTACGCC

> sq5;size=306854;Mn-incubations;Sva1033;

TACGGAGGGTGCAAACGTTGTTCGGAATTATTGGGCGTAAAGAGCATGTAGGCGGT
CTGTCAAGTCTGATGTGAAAGCCCGGGGCTCAACCCCGGAAGTGCATTGGAAACTG
GCAGACTTGAGTACGGGAGAGGAAAGTGGAATTTCGAGTGTAGGGGTGAAATCCGT
AGATATTCGAAGGAACACCAGTGGCGAAGGCGGCTTTCTGGACCGATACTGACGCT
GAGATGCGAAAGCGTGGGGAGCAAACA

> sq28;size=45573;Mn-incubations;Sva1033;

TACGGAGGGTGCAAGCGTTGTTCGGAATTATTGGGCGTAAAGAGCATGTAGGCGGC
TCGCCAAGTCTGATGTGAAAGCCCTGGGCTCAACCCAGGAAGTGCATTGGAAACTG
GCGAACTTGAGTACGGGAGAGGAAAGTGGAATTTCGAGTGTAGGGGTGAAATCCGT
AGATATTCGAAGGAACACCAGTGGCGAAGGCGGCTTTCTGGACCGATACTGACGCT
GAGATGCGAAAGCGTGGGGAGCAAACA

> sq33;size=37894;Mn-incubations;Sva1033;

TACGGAGGGTGCAAGCGTTGTTCGGAATTATTGGGCGTAAAGAGCGTGTAGGCGGC
TCGCCAAGTCTGATGTGAAAGCCCTGGGCTCAACCCAGGAAGTGCATTGGAAACTG
GCGAACTTGAGTACGGGAGAGGAAAGTGGAATTTCGAGTGTAGGGGTGAAATCCGT
AGATATTCGAAGGAACACCAGTGGCGAAGGCGGCTTTCTGGACCGATACTGACGCT
GAGACGCGAAAGCGTGGGGAGCAAACA

1.5 Calculation of solid phase manganese data

Solid phase manganese was extracted and quantified by Monien et al. (2014a) and the data published under doi.pangaea.de/10.1594/PANGAEA.805935 (Monien et al., 2014b). The station P01 sampled by Monien et al. (2014a) was in close proximity to STA01 sampled in this study (Figure S 9A). However, the data was published as wt.%. In order to compare the manganese content to other studies, we converted MnO (salt corrected wt.%) into MnO (µmol/cm³) by the calculations below. The used water content values were also published in Monien et al. (2014b). The density of dry sediment was assumed as 2.6 g/cm³ and the density of water as 1 g/cm³.

[1] $$MnO\left[\frac{mg}{g}\right] = MnO\,[wt.\%] \times 10$$

[2] $$MnO\left[\frac{\mu mol}{g\ dry\ sediment}\right] = \frac{MnO\,[\frac{mg}{g}]}{M(MnO)\,[\frac{mg}{mmol}]} \times 1000$$

[3] $$V_{dry\ sediment}\left[\frac{cm^3}{1\ g\ wet\ sediment}\right] = \frac{0.01 \times (100 - water\ content\,[\%])}{density_{dry\ sediment}\left[\frac{g}{cm^3}\right]}$$

[4] $$V_{water}\left[\frac{cm^3}{1\ g\ wet\ sediment}\right] = \frac{0.01 \times water\ content\,[\%]}{density_{water}\left[\frac{g}{cm^3}\right]}$$

[5] $$V_{dry\ sediment}\left[\frac{cm^3}{1\ cm^3\ wet\ sediment}\right] = \frac{V_{dry\ sediment}\left[\frac{cm^3}{1\ g\ wet\ sediment}\right] \times 1\,[cm^3\ wet\ sediment]}{V_{dry\ sediment}\left[\frac{cm^3}{1\ g\ wet\ sediment}\right] + V_{water}\left[\frac{cm^3}{1\ g\ wet\ sediment}\right]}$$

[6] $$m_{dry\ sediment}\left[\frac{g}{1\ cm^3\ wet\ sediment}\right] = V_{dry\ sediment}\left[\frac{cm^3}{1\ cm^3\ wet\ sediment}\right] \times density_{dry\ sediment}\left[\frac{g}{cm^3}\right]$$

[7] $$Mn\left[\frac{\mu mol}{cm^3}\right] = MnO\left[\frac{\mu mol}{g\ dry\ sediment}\right] \times m_{dry\ sediment}\left[\frac{g}{1\ cm^3\ wet\ sediment}\right]$$

The R code used for the calculations is also published in the Github repository https://github.com/Microbial-Ecophysiology/Mn-red-PotterCove.

2 Supplementary figures and tables

2.1 Supplementary figures

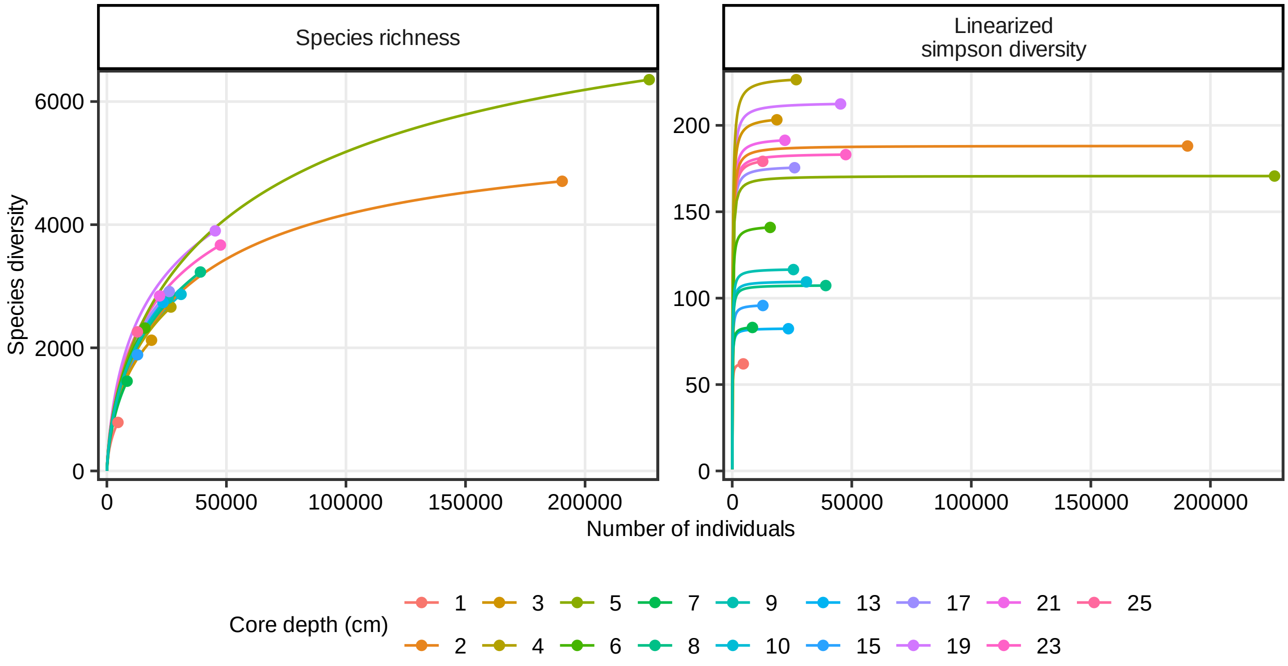

Figure S1: Rarefaction curves of 16S rRNA gene amplicon sequencing of *in situ* sediment core STA01.02.

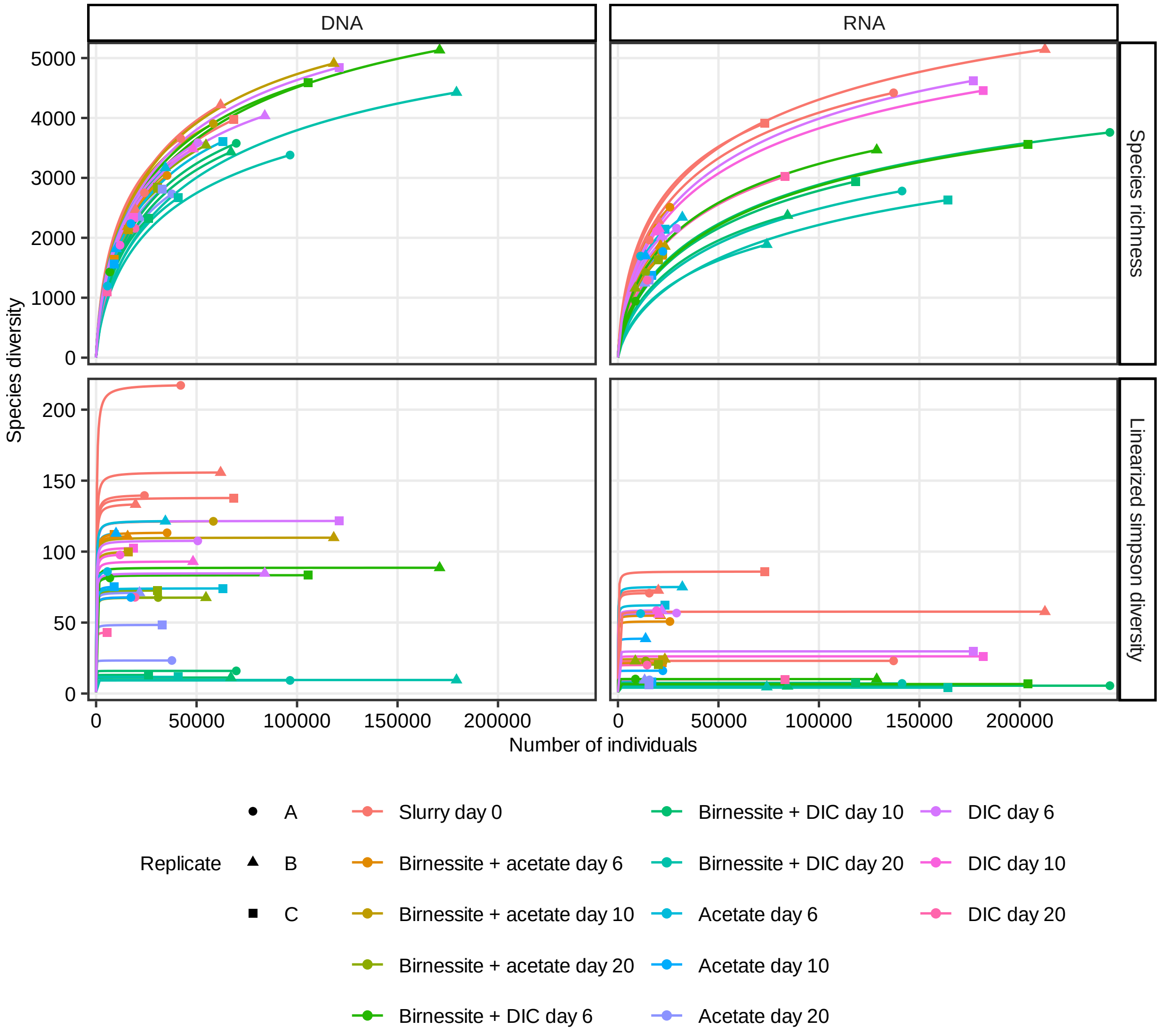

Figure S2: Rarefaction curves of 16S rRNA gene (DNA) and 16S rRNA (RNA) amplicon sequencing of incubation experiment.

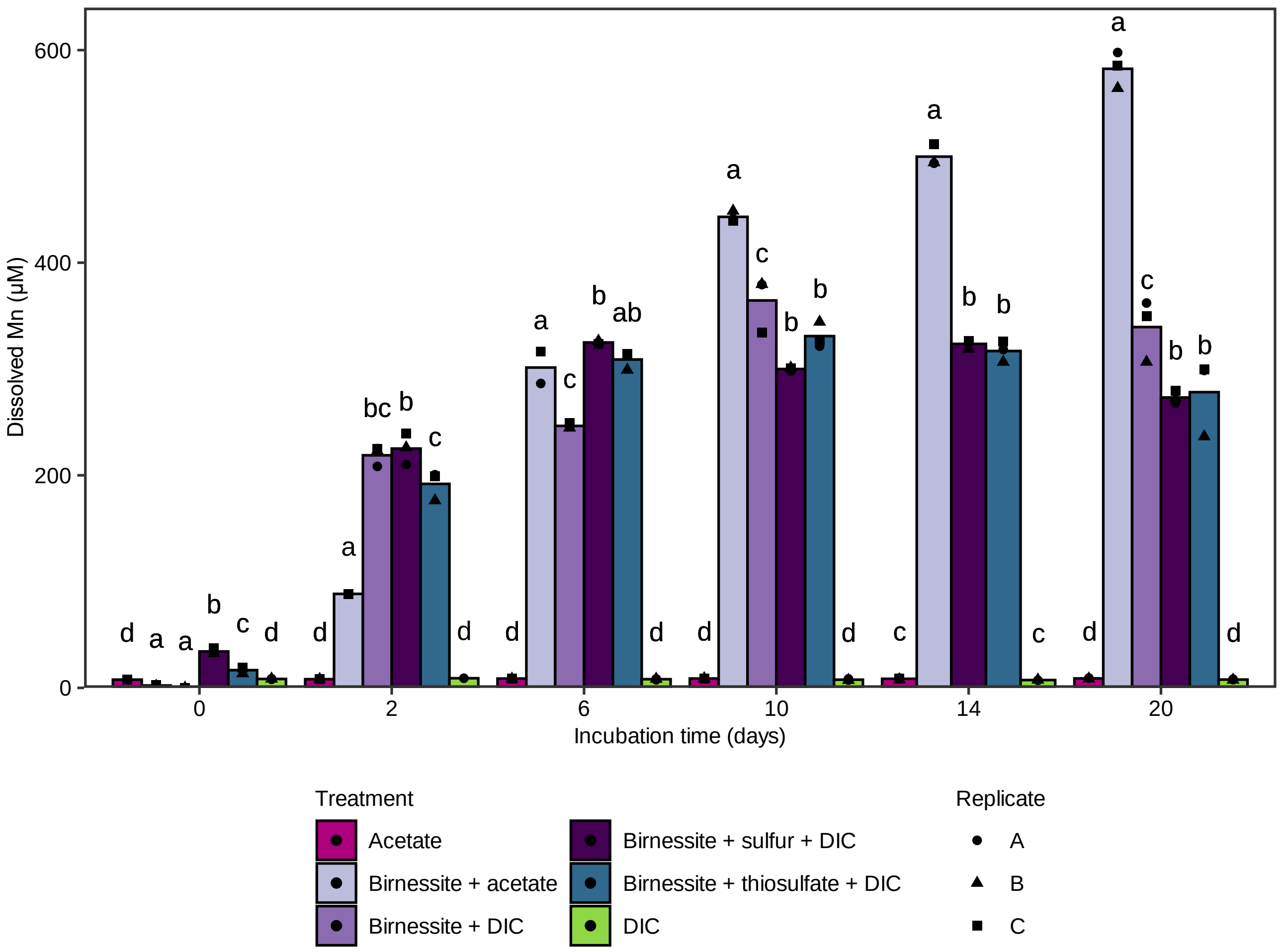

Figure S3: Concentration of dissolved Mn in slurry incubations over the incubation time. The bars represent calculated means per treatment distinguished by color, individual points are displayed on top distinguished by shape. General linear hypothesis for multiple comparisons were performed within each time point and different letters represent statistically significant differences ($p < 0.05$) between treatments.

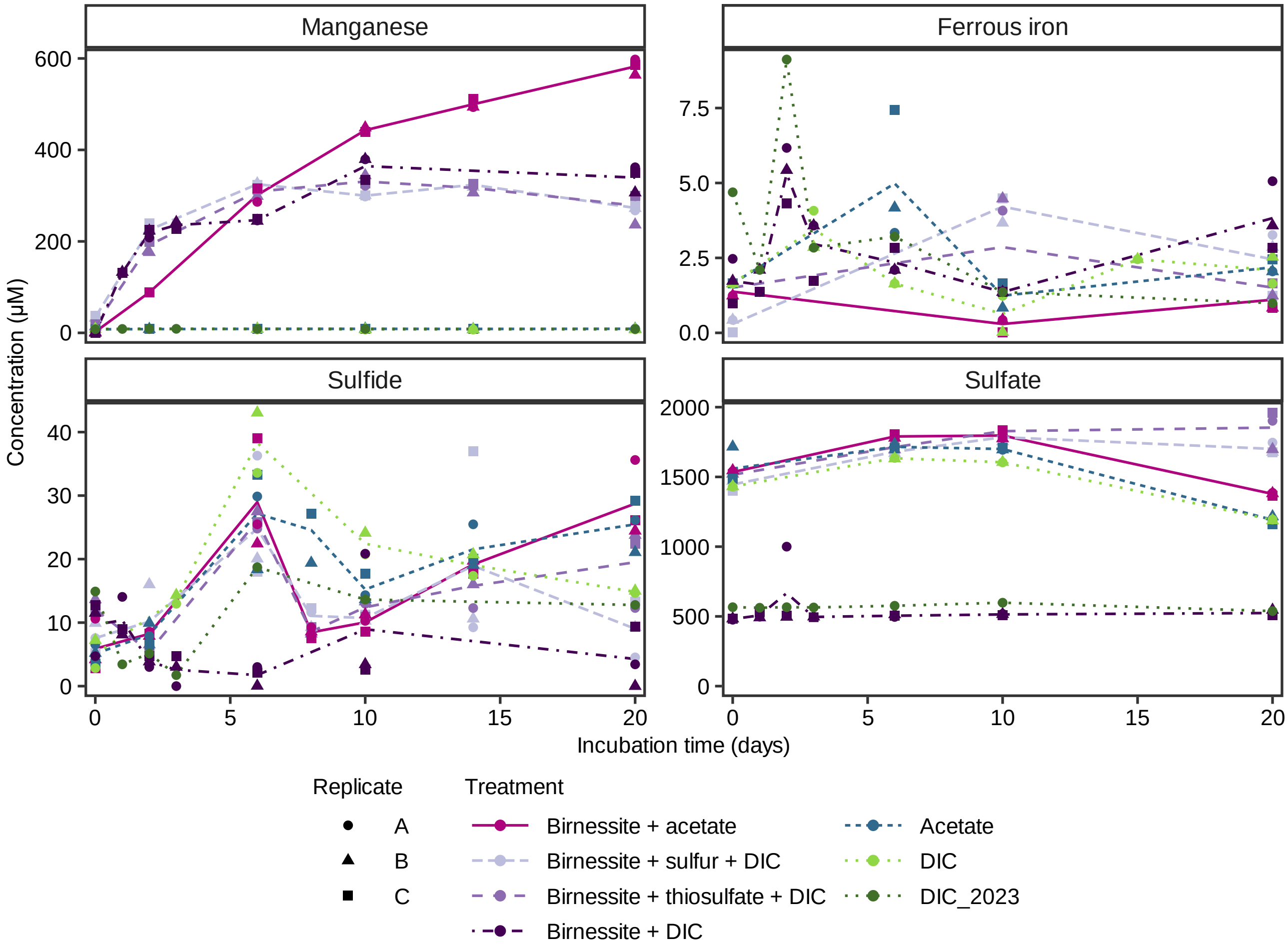

Figure S4: Geochemistry in aqueous slurry phase over time. Incubations Birnessite + DIC and DIC_2023 were run in spring 2023 while all other treatments were run in summer 2021. Likely due to longer storage, the sulfate concentration in the 2023 run treatments were lower (see text for more details).

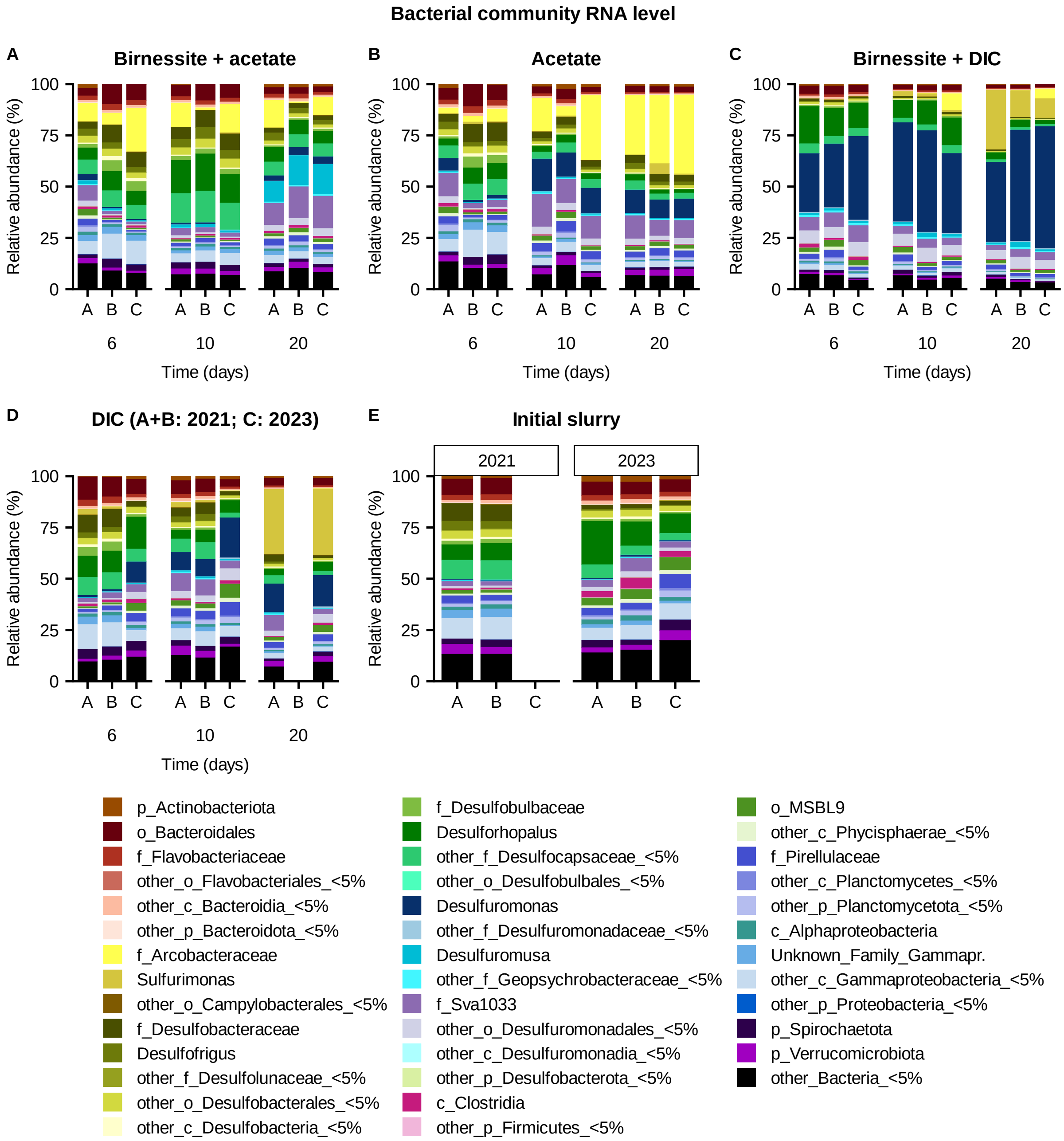

Figure S5: Bacterial 16S rRNA community of incubation experiment. Incubations (A) Birnessite + acetate and (B) Acetate were performed in 2021, incubation (C) Birnessite + DIC was performed in 2023. For both set-ups a control incubation (D) with only DIC was performed, in duplicates for 2021 (replicate A, B) and in single set-up for 2023 (replicate C). For both set-ups the initial slurry of day 0 was sequenced (E) in duplicates for 2021, in triplicates for 2023. All other treatments were performed in triplicate incubation bottles (A, B, C). Plotted is the whole community with genera above 5% relative abundance in at least one sample. If a genus was not above the threshold or not classified on this level, the next higher rank was plotted if it was above the threshold and so on.

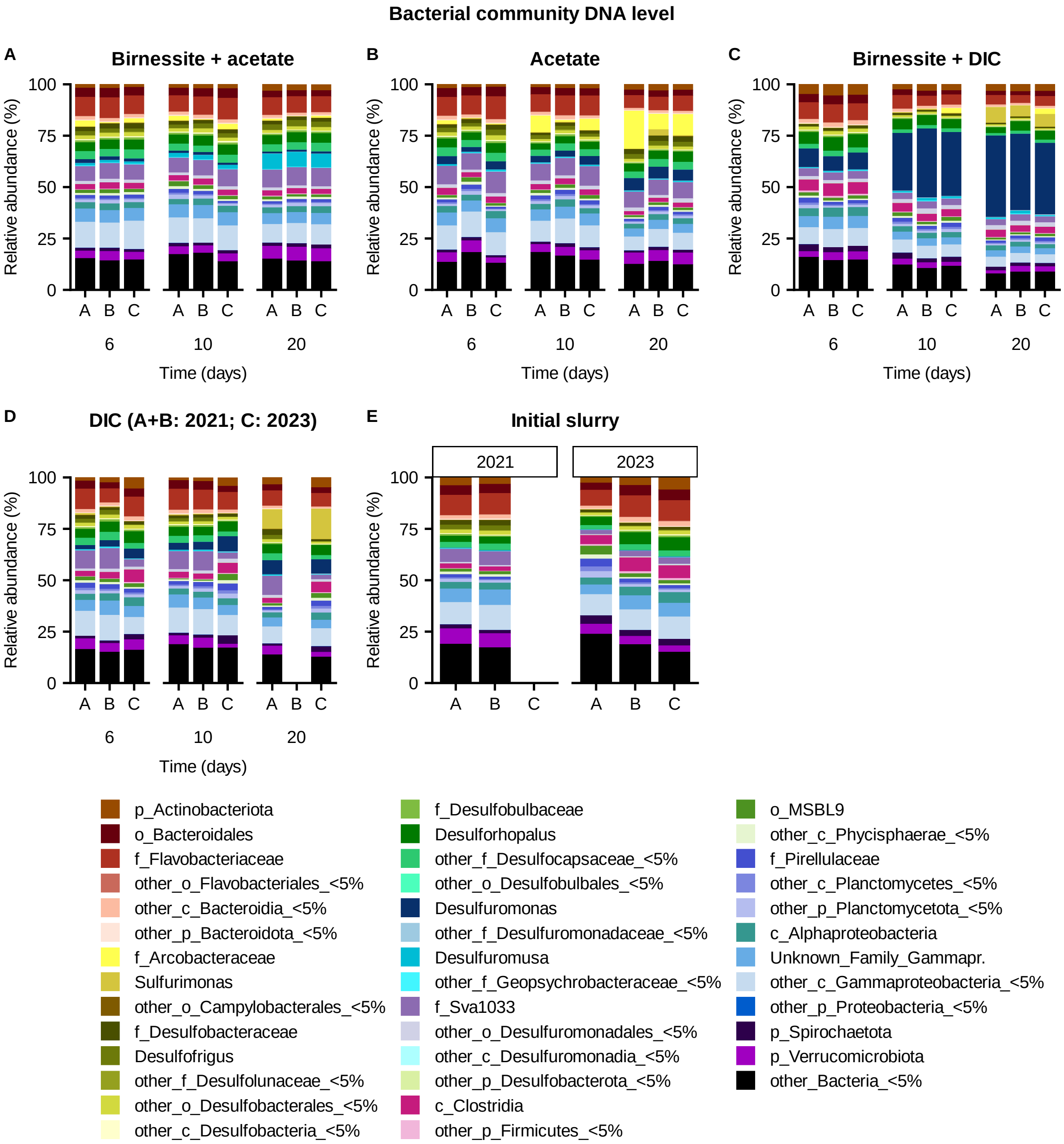

Figure S6: Bacterial 16S rRNA gene community of incubation experiment. Incubations (A) Birnessite + acetate and (B) Acetate were performed in 2021, incubation (C) Birnessite + DIC was performed in 2023. For both set-ups a control incubation (D) with only DIC was performed, in duplicates for 2021 (replicate A, B) and in single set-up for 2023 (replicate C). For both set-ups the initial slurry of day 0 was sequenced (E) in duplicates for 2021, in triplicates for 2023. All other treatments were performed in triplicate incubation bottles (A, B, C). Plotted is the whole community with genera above 5% relative abundance in at least one sample. If a genus was not above the threshold or not classified on this level, the next higher rank was plotted if it was above the threshold and so on.

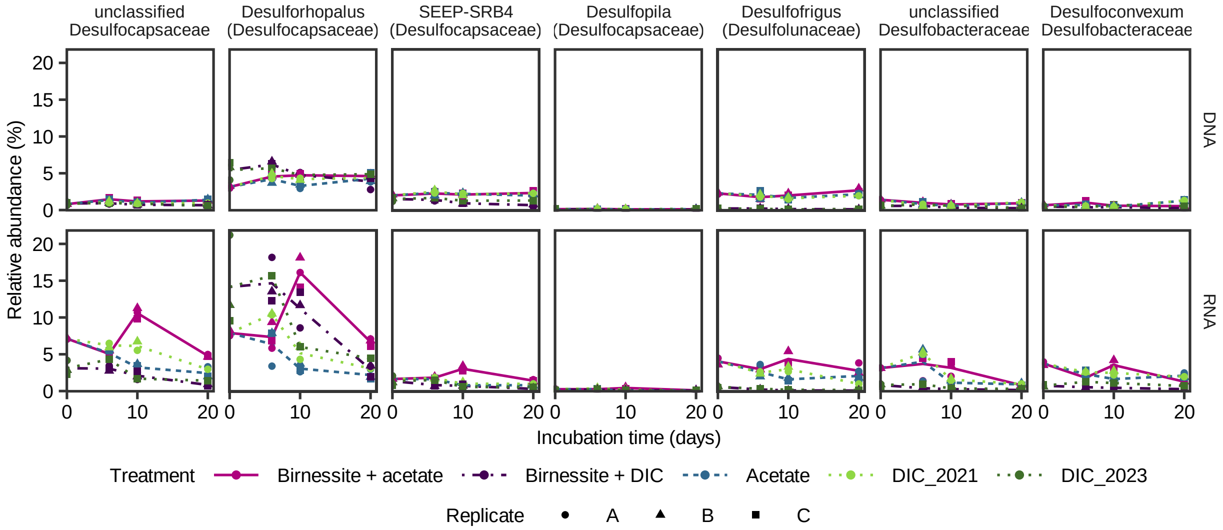

Figure S7: Relative abundance of 16S rRNA gene (top row DNA) and 16S rRNA (bottom row RNA) of selected taxa. Displayed are the same taxonomic groups plotted in Figure S 5 and Figure S 6 in a higher taxonomic resolution. Sample of day 0 was sequenced from duplicate incubation bottles for 2021 incubations while all other time points were sequenced from triplicate incubation bottles separately for each treatment. Different treatments are displayed by color and line type, and incubation triplicates by shape. The lines connect calculated means per treatment. DIC control treatments for 2021 and 2023 are plotted separately.

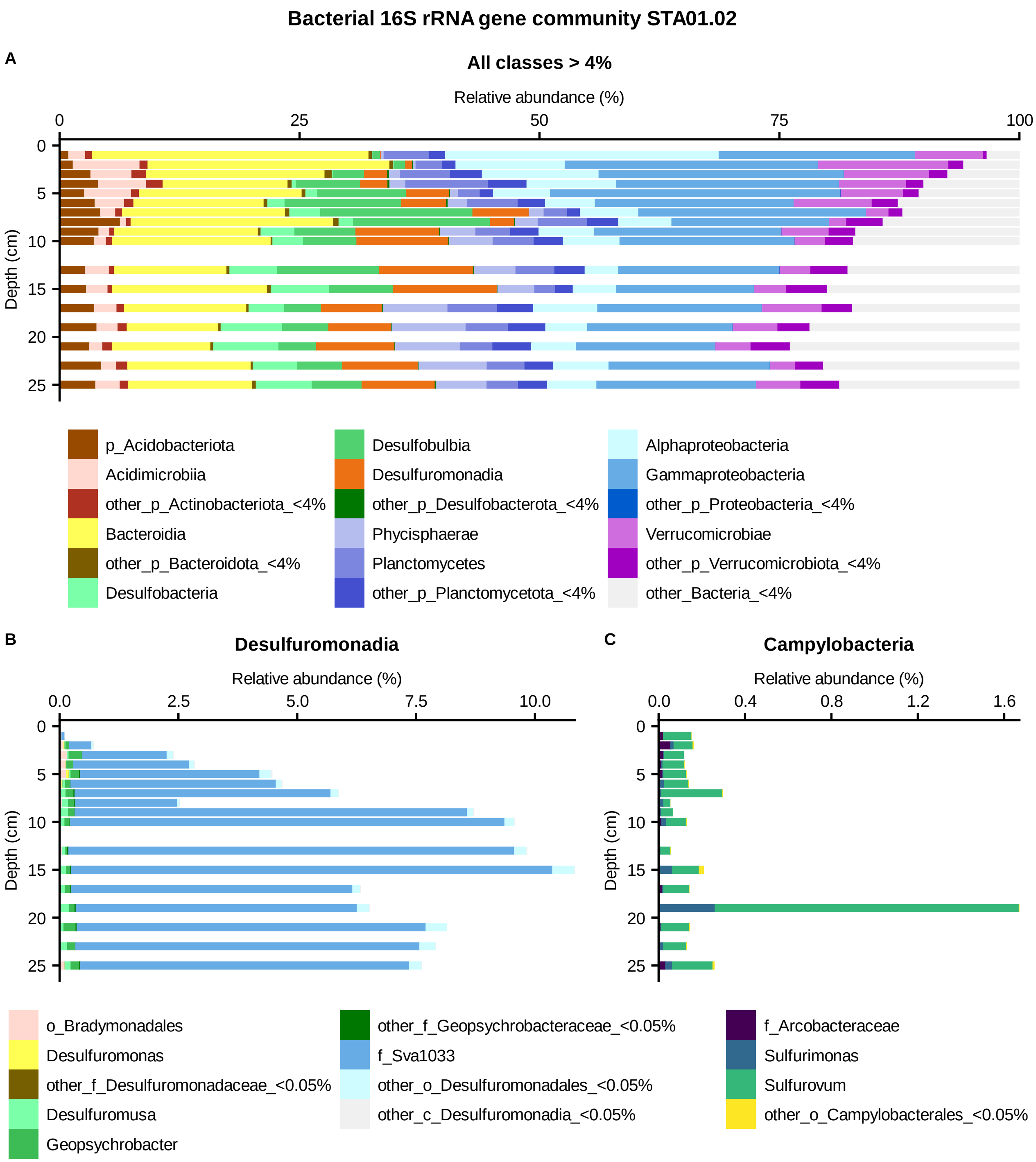

Figure S8: *In situ* bacterial community based on 16S rRNA gene sequencing of core STA01.02 used for incubation set-up. Plotted are (A) the whole community with classes above 4% relative abundance in at least one sample, (B) genera of *Desulfuromonadia* and (C) *Campylobacteria*. Note the different x-scales.

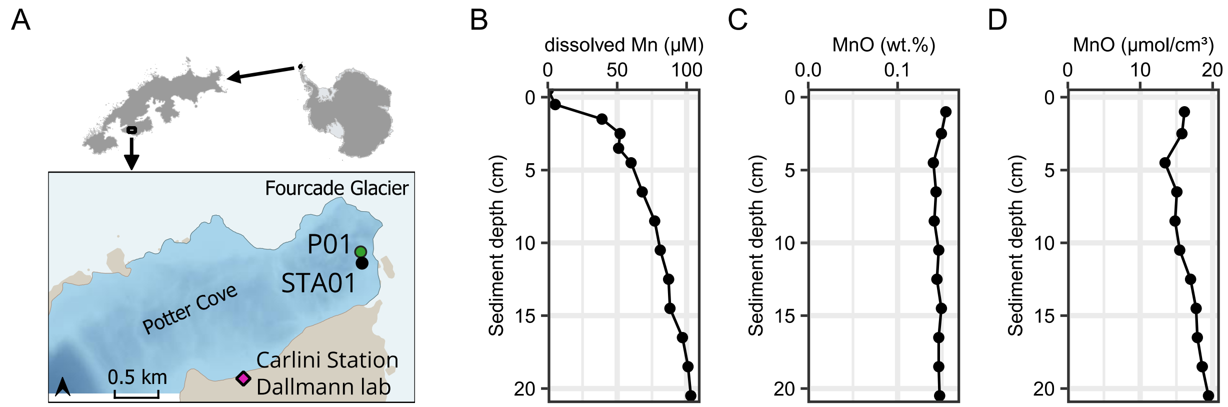

Figure S9: Manganese oxides and dissolved manganese in sediments at site PC-P01 (Monien et al., 2014a), which is near STA01 of this study (A). Data replotted from Monien et al. (2014b) dissolved Mn in pore water (B), solid phase MnO in wt.%, salt corrected (C) and recalculated into µmol/cm³, using provided water content and literature values for sediment density, for comparison with other datasets (D), see text for details. Map created with QGIS 3.34.3, bathymetry data Neder et al. (2022) updated from Jerosch et al. (2015), basemap data SCAR Antarctic Digital Database 2023, rock outcrop from Gerrish (2020) manually smoothed.

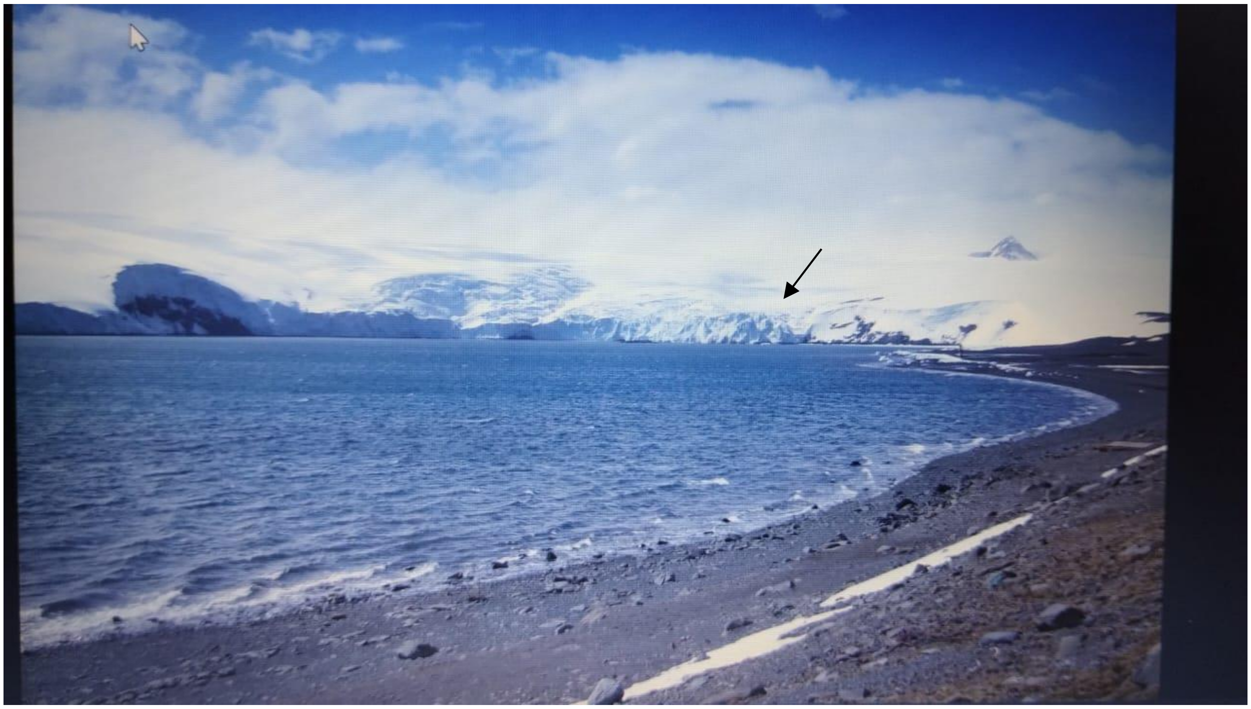

Figure S10: Photo Potter Cove Fourcade glacier 2011 with permission by M. Sierra. Location STA 01 indicated by arrow.

2.2 Supplementary tables

Table S1: Trimming parameters used during sequence analysis for samples sequenced in different libraries. Some samples were sequenced in multiple libraries and flowcells in order to retrieve sufficient reads.

**Samples**	**Lib ID**	**Flowcell lane ID**	**Trimming**		**Sequence lengths kept (bp)**
			**R1 length (bp)**	**R2 length (bp)**	
**Incubation experiment**	Lib22	HKMLNDRXY_L1	120	170	249-254, 300
	Lib23	HKMLNDRXY_L1	120	170	249-254, 300
	Lib87	H7H5VDRX5_L1	110	180	249-254, 300
	Lib87	H7JJGDRX5_L1	110	180	249-254, 300
**in situ**	Lib5	HHMW5DRXX_L1	90	200	249-254, 276
	Lib5	HJ72JDRXX_L2	90	200	249-254, 276
	Lib45	H7W7NDRX2_L2	120	170	249-254, 276
	Lib45	H7WKNDRX2_L2	110	180	249-254, 276

Table S2: BLAST results for most abundant ASVs of *Arcobacteraceae* in incubation experiment

Query ASV	Description	Scientific Name	Max Score	Total Score	Query Cover	E value	Per. ident	Acc. Len	Accession no.	origin	potential linked publication
sq10	Uncultured bacterium clone EB1_3cmSIP-B50 16S ribosomal RNA gene	uncultured bacterium	453	453	100%	6.E-123	100	897	MK108086.1	Ulleung Basin, East Sea	(Cho et al., 2020)
	Uncultured bacterium clone SS1_B_02_37 16S ribosomal RNA gene	uncultured bacterium	449	449	100%	3.E-121	99.6	1389	EU050947.1	Kings Bay Svalbard	
	Arcobacteraceae bacterium strain IMCC39198 16S ribosomal RNA gene	Arcobacteraceae bacterium	435	435	100%	2.E-117	98.41	1318	OQ807895.1	tidal flat sediment, South Korea Ganghwa island	
	Arcobacter sp. HL735A partial 16S rRNA gene	Arcobacter sp. HL735A	426	426	100%	9.E-115	97.61	1378	LR722870.1	coastal marine surface water, where?	
	Poseidonibacter ostreae strain SJOD-M-33 16S ribosomal RNA gene	Poseidonibacter ostreae	426	426	100%	9.E-115	97.61	1517	MN549520.1	gut of Ostrea, Seomjin River	(Baek et al., 2023)
	Arcobacter sp. strain s4j51-2 16S ribosomal RNA gene	Arcobacter sp.	426	426	100%	9.E-115	97.61	1478	MK140991.1	no info	
	Arcobacter sp. strain s4j41-3 16S ribosomal RNA gene	Arcobacter sp.	426	426	100%	9.E-115	97.61	1478	MK140989.1	no info	
	Poseidonibacter antarcticus strain SM1702 16S ribosomal RNA gene	Poseidonibacter antarcticus	426	426	100%	9.E-115	97.61	1478	MH473590.1	Antarctic intertidal sediment off Ardely Island, West Antarctica	(Guo et al., 2019)
	Halarcobacter bivalviorum strain D-5 16S ribosomal RNA gene	Halarcobacter bivalviorum	417	417	100%	5.E-112	96.81	808	MT254910.1	mussels Ebro Delta	(Miller et al., 2018)
	Arcobacter arenosus strain CAU 1517 16S ribosomal RNA gene	Arcobacter arenosus	417	417	100%	5.E-112	96.81	1482	MK280766.1	marine sediment Busan Korea	

Query ASV	Description	Scientific Name	Max Score	Total Score	Query Cover	E value	Per. ident	Acc. Len	Accession no.	origin	potential linked publication
sq23	Uncultured bacterium clone SS1_B_02_37 16S ribosomal RNA gene	uncultured bacterium	453	453	100%	7.E-123	100	1389	EU050947.1	Kings Bay Svalbard	
	Uncultured bacterium clone EB1_3cmSIP-B50 16S ribosomal RNA gene	uncultured bacterium	449	449	100%	3.E-121	99.6	897	MK108086.1	Ulleung Basin, East Sea	
	Arcobacteraceae bacterium strain IMCC39198 16S ribosomal RNA gene	Arcobacteraceae bacterium	431	431	100%	8.E-116	98.01	1318	OQ807895.1	tidal flat sediment, South Korea Ganghwa island	
	Arcobacter sp. HL735A partial 16S rRNA gene	Arcobacter sp. HL735A	422	422	100%	4.E-113	97.21	1378	LR722870.1	coastal marine surface water, where?	
	Poseidonibacter ostreae strain SJOD-M-33 16S ribosomal RNA gene	Poseidonibacter ostreae	422	422	100%	4.E-113	97.21	1517	MN549520.1	gut of Ostrea, Seomjin River	(Baek et al., 2023)
	Arcobacter sp. strain s4j51-2 16S ribosomal RNA gene	Arcobacter sp.	422	422	100%	4.E-113	97.21	1478	MK140991.1	no info	
	Arcobacter sp. strain s4j41-3 16S ribosomal RNA gene	Arcobacter sp.	422	422	100%	4.E-113	97.21	1478	MK140989.1	no info	
	Poseidonibacter antarcticus strain SM1702 16S ribosomal RNA gene	Poseidonibacter antarcticus	422	422	100%	4.E-113	97.21	1478	MH473590.1	Antarctic intertidal sediment off Ardely Island, West Antarctica	(Guo et al., 2019)
	Halarcobacter bivalviorum strain D-5 16S ribosomal RNA gene	Halarcobacter bivalviorum	413	413	100%	2.E-110	96.41	808	MT254910.1	mussels Ebro Delta	(Miller et al., 2018)
	Arcobacter arenosus strain CAU 1517 16S ribosomal RNA gene	Arcobacter arenosus	413	413	100%	2.E-110	96.41	1482	MK280766.1	marine sediment Busan Korea	

Query ASV	Description	Scientific Name	Max Score	Total Score	Query Cover	E value	Per. ident	Acc. Len	Accession no.	origin	potential linked publication
sq60	Arcobacteraceae bacterium strain IMCC39198 16S ribosomal RNA gene	Arcobacteraceae bacterium	453	453	100%	7.E-123	100	1318	OQ807895.1	tidal flat sediment, South Korea Ganghwa island	
	Uncultured Arcobacter sp. clone OTU_A7_SP1_98 16S ribosomal RNA gene	uncultured Arcobacter sp.	449	449	100%	3.E-121	99.6	696	JF928644.1	microplastic surface, coastal marine sediment	(Harrison et al., 2014)
	Arcobacter sp. HL735A partial 16S rRNA gene	Arcobacter sp. HL735A	444	444	100%	3.E-120	99.2	1378	LR722870.1	coastal marine surface water, where?	
	Poseidonibacter ostreae strain SJOD-M-33 16S ribosomal RNA gene	Poseidonibacter ostreae	444	444	100%	3.E-120	99.2	1517	MN549520.1	gut of Ostrea, Seomjin River	(Baek et al., 2023)
	Uncultured bacterium clone EB1_3cmSIP-B50 16S ribosomal RNA gene	uncultured bacterium	435	435	100%	2.E-117	98.41	897	MK108086.1	Ulleung Basin, East Sea	
	Arcobacter sp. strain s4j51-2 16S ribosomal RNA gene	Arcobacter sp.	431	431	100%	8.E-116	98.01	1478	MK140991.1	no info	
	Arcobacter sp. strain s4j41-3 16S ribosomal RNA gene	Arcobacter sp.	431	431	100%	8.E-116	98.01	1478	MK140989.1	no info	
	Poseidonibacter antarcticus strain SM1702 16S ribosomal RNA gene	Poseidonibacter antarcticus	431	431	100%	8.E-116	98.01	1478	MH473590.1	Antarctic intertidal sediment off Ardely Island, West Antarctica	(Guo et al., 2019)

Table S3: BLAST results for most abundant ASVs of *Desulfuromonas* in incubation experiment

Query ASV	Description	Scientific Name	Max Score	Total Score	Query Cover	E value	Per. ident	Acc. Len	Accession no.	origin	potential linked publication
sq4	Uncultured bacterium clone GAE_41 16S ribosomal RNA gene	uncultured bacterium	453	453	100%	7.E-123	100	799	KF623796.1	sulfur-precipitating mat, Cathedral Hill hydrothermal venting site, Guaymas Basin	(Pjevac et al., 2014)
	Desulfuromonas svalbardensis strain 112 16S ribosomal RNA	Desulfuromonas svalbardensis	453	453	100%	7.E-123	100	1516	NR_043213.1	Arctic marine sediment	(Vandieken et al., 2006)
	Desulfuromonas svalbardensis strain 60 16S ribosomal RNA gene	Desulfuromonas svalbardensis	449	449	100%	3.E-121	99.6	1516	AY835390.1	Arctic marine sediment	(Vandieken et al., 2006)
	Desulfuromonas acetoxidans strain DSM 684 16S ribosomal RNA	Desulfuromonas acetoxidans	444	444	100%	3.E-120	99.2	1558	NR_121678.1	Antarctic sediment South Orkney Islands	(Pfennig and Biebl, 1976)
	Desulfuromonas svalbardensis strain 103 16S ribosomal RNA gene	Desulfuromonas svalbardensis	444	444	100%	3.E-120	99.2	1513	AY835391.1	Arctic marine sediment	(Vandieken et al., 2006)
	Desulfuromonas svalbardensis strain 49 16S ribosomal RNA gene	Desulfuromonas svalbardensis	444	444	100%	3.E-120	99.2	1514	AY835389.1	Arctic marine sediment	(Vandieken et al., 2006)
sq1	Desulfuromonas svalbardensis strain 103 16S ribosomal RNA gene	Desulfuromonas svalbardensis	453	453	100%	7.E-123	100	1513	AY835391.1	Arctic marine sediment	(Vandieken et al., 2006)
	Desulfuromonas svalbardensis strain 49 16S ribosomal RNA gene	Desulfuromonas svalbardensis	453	453	100%	7.E-123	100	1514	AY835389.1	Arctic marine sediment	(Vandieken et al., 2006)
	Desulfuromonas svalbardensis strain 60 16S ribosomal RNA gene	Desulfuromonas svalbardensis	449	449	100%	3.E-121	99.6	1516	AY835390.1	Arctic marine sediment	(Vandieken et al., 2006)
	Uncultured bacterium clone GAE_41 16S ribosomal RNA gene	uncultured bacterium	444	444	100%	3.E-120	99.2	799	KF623796.1	sulfur-precipitating mat, Cathedral Hill hydrothermal venting site, Guaymas Basin	(Pjevac et al., 2014)
	Desulfuromonas svalbardensis strain 112 16S ribosomal RNA	Desulfuromonas svalbardensis	444	444	100%	3.E-120	99.2	1516	NR_043213.1	Arctic marine sediment	(Vandieken et al., 2006)

Query ASV	Description	Scientific Name	Max Score	Total Score	Query Cover	E value	Per. ident	Acc. Len	Accession no.	origin	potential linked publication
sq18	Desulfuromonas svalbardensis strain 60 16S ribosomal RNA gene	Desulfuromonas svalbardensis	453	453	100%	7.E-123	100	1516	AY835390.1	Arctic marine sediment	(Vandieken et al., 2006)
	Uncultured bacterium clone GAE_41 16S ribosomal RNA gene	uncultured bacterium	449	449	100%	3.E-121	99.6	799	KF623796.1	sulfur-precipitating mat, Cathedral Hill hydrothermal venting site, Guaymas Basin	(Pjevac et al., 2014)
	Desulfuromonas svalbardensis strain 112 16S ribosomal RNA	Desulfuromonas svalbardensis	449	449	100%	3.E-121	99.6	1516	NR_043213.1	Arctic marine sediment	(Vandieken et al., 2006)
	Desulfuromonas svalbardensis strain 103 16S ribosomal RNA gene	Desulfuromonas svalbardensis	449	449	100%	3.E-121	99.6	1513	AY835391.1	Arctic marine sediment	(Vandieken et al., 2006)
	Desulfuromonas svalbardensis strain 49 16S ribosomal RNA gene	Desulfuromonas svalbardensis	449	449	100%	3.E-121	99.6	1514	AY835389.1	Arctic marine sediment	(Vandieken et al., 2006)
sq19	Desulfuromonas svalbardensis strain 103 16S ribosomal RNA gene	Desulfuromonas svalbardensis	449	449	100%	3.E-121	99.6	1513	AY835391.1	Arctic marine sediment	(Vandieken et al., 2006)
	Desulfuromonas svalbardensis strain 49 16S ribosomal RNA gene	Desulfuromonas svalbardensis	449	449	100%	3.E-121	99.6	1514	AY835389.1	Arctic marine sediment	(Vandieken et al., 2006)
	Uncultured bacterium clone GAE_41 16S ribosomal RNA gene	uncultured bacterium	444	444	100%	3.E-12	99.2	799	KF623796.1	sulfur-precipitating mat, Cathedral Hill hydrothermal venting site, Guaymas Basin	(Pjevac et al., 2014)
	Desulfuromonas svalbardensis strain 112 16S ribosomal RNA	Desulfuromonas svalbardensis	444	444	100%	3.E-12	99.2	1516	NR_043213.1	Arctic marine sediment	(Vandieken et al., 2006)
	Desulfuromonas svalbardensis strain 60 16S ribosomal RNA gene	Desulfuromonas svalbardensis	444	444	100%	3.E-12	99.2	1516	AY835390.1	Arctic marine sediment	(Vandieken et al., 2006)

Table S4: BLAST results for most abundant ASVs of *Desulfuromusa* in incubation experiment

Query ASV	Description	Scientific Name	Max Score	Total Score	Query Cover	E value	Per. ident	Acc. Len	Accession no.	origin	potential linked publication
sq22	Desulfuromusa sp. Fe30-7C-S gene for 16S rRNA	Desulfuromusa sp. Fe30-7C-S	453	453	100%	7.E-123	100	1150	AB304907.1	black smoker chimney North Slope field Brothers Caldera Kermadec Arc	(Takai et al., 2009)
	Desulfuromusa bakii strain Gyprop 16S ribosomal RNA	Desulfuromusa bakii	449	449	100%	3.E-121	99.6	1473	NR_026175.1	Guayamas Basin	(Liesack and Finster, 1994)
	Bacterium N05X partial 16S rRNA gene	bacterium N05X	440	440	100%	1.E-118	98.8	692	AJ786071.1	tidal flat surface sediment, North Sea	(Köpke et al., 2005)
	Malonomonas rubra strain GraMal1 16S ribosomal RNA	Malonomonas rubra	440	440	100%	1.E-118	98.8	1525	NR_026479.1	mud Canal Grande Venice	(Dehning and Schink, 1989)

Table S5: BLAST results for most abundant ASVs of Sva1033 in incubation experiment

Query ASV	Description	Scientific Name	Max Score	Total Score	Query Cover	E value	Per. ident	Acc. Len	Accession no.	origin	potential linked publication
sq5	Deltaproteobacteria bacterium strain IMCC39542 16S ribosomal RNA gene	Deltaproteobacteria bacterium	453	453	100%	7.E-123	100	1372	OQ808218.1	tidal flat sediment, South Korea Ganghwa island	
	Deltaproteobacteria bacterium strain IMCC39489 16S ribosomal RNA gene	Deltaproteobacteria bacterium	453	453	100%	7.E-123	100	1478	OQ808176.1	tidal flat sediment, South Korea Ganghwa island	
	Pelobacter sp. 16S rRNA gene	Pelobacter sp. A3b3	453	453	100%	7.E-123	100	1551	AJ271656.1	Black Sea shelf sediments	(Thamdrup et al., 2000)
	Deltaproteobacteria bacterium strain IMCC39461 16S ribosomal RNA gene	Deltaproteobacteria bacterium	413	413	100%	2.E-110	96.41	1374	OQ808150.1	tidal flat sediment, South Korea Ganghwa island	
	Deltaproteobacteria bacterium strain IMCC39484 16S ribosomal RNA gene	Deltaproteobacteria bacterium	413	413	100%	2.E-110	96.41	1428	OQ808171.1	tidal flat sediment, South Korea Ganghwa island	
	Desulfuromonas acetoxidans strain DSM 684 16S ribosomal RNA	Desulfuromonas acetoxidans	408	408	100%	3.E-109	96.02	1558	NR_121678.1	Antarctic sediment South Orkney Islands	(Pfennig and Biebl, 1976)
	Delta proteobacterium S1 16S ribosomal RNA gene	Desulfuromusa sp. S1	408	408	100%	3.E-109	96.02	1436	AY187309.1	current-harvesting electrode in marine fuel cell	(Holmes et al., 2004)
	Delta Proteobacterium G50VI partial 16S rRNA gene	delta proteobacterium G50VI	404	404	100%	1.E-107	95.62	705	AJ786070.1	subsurface sediment tidal flat; North Sea	(Köpke et al., 2005)
	Deltaproteobacteria bacterium strain IMCC39547 16S ribosomal RNA gene	Deltaproteobacteria bacterium	399	399	100%	1.E-106	95.22	1411	OQ808222.1	tidal flat sediment, South Korea Ganghwa island	

Query ASV	Description	Scientific Name	Max Score	Total Score	Query Cover	E value	Per. ident	Acc. Len	Accession no.	origin	potential linked publication
sq28	Deltaproteobacteria bacterium strain IMCC39461 16S ribosomal RNA gene	Deltaproteobacteria bacterium	453	453	100%	7.E-123	100	1374	OQ808150.1	tidal flat sediment, South Korea Ganghwa island	
	Deltaproteobacteria bacterium strain IMCC39547 16S ribosomal RNA gene	Deltaproteobacteria bacterium	440	440	100%	1.E-118	98.8	1411	OQ808222.1	tidal flat sediment, South Korea Ganghwa island	
	Deltaproteobacteria bacterium strain IMCC39484 16S ribosomal RNA gene	Deltaproteobacteria bacterium	426	426	100%	9.E-115	97.61	1428	OQ808171.1	tidal flat sediment, South Korea Ganghwa island	
	Deltaproteobacteria bacterium strain IMCC39542 16S ribosomal RNA gene	Deltaproteobacteria bacterium	413	413	100%	2.E-110	96.41	1372	OQ808218.1	tidal flat sediment, South Korea Ganghwa island	
	Deltaproteobacteria bacterium strain IMCC39489 16S ribosomal RNA gene	Deltaproteobacteria bacterium	413	413	100%	2.E-110	96.41	1478	OQ808176.1	tidal flat sediment, South Korea Ganghwa island	
	Pelobacter sp. 16S rRNA gene	Pelobacter sp. A3b3	413	413	100%	2.E-110	96.41	1551	AJ271656.1	Black Sea shelf sediments	(Thamdrup et al., 2000)
	Delta Proteobacterium G50VI partial 16S rRNA gene	delta proteobacterium G50VI	408	408	100%	3.E-109	96.02	705	AJ786070.1	subsurface sediment tidal flat; North Sea	(Köpke et al., 2005)
	Desulfuromonas acetoxidans strain DSM 684 16S ribosomal RNA	Desulfuromonas acetoxidans	404	404	100%	1.E-107	95.62	1558	NR_121678.1	Antarctic sediment South Orkney Islands	(Pfennig and Biebl, 1976)
	Delta proteobacterium S1 16S ribosomal RNA gene	Desulfuromusa sp. S1	377	377	100%	1.E-99	93.23	1436	AY187309.1	current-harvesting electrode in marine fuel cell	(Holmes et al., 2004)

Query ASV	Description	Scientific Name	Max Score	Total Score	Query Cover	E value	Per. ident	Acc. Len	Accession no.	origin	potential linked publication
sq33	Deltaproteobacteria bacterium strain IMCC39547 16S ribosomal RNA gene	Deltaproteobacteria bacterium	449	449	100%	3.E-121	99.6	1411	OQ808222.1	tidal flat sediment, South Korea Ganghwa island	
	Deltaproteobacteria bacterium strain IMCC39461 16S ribosomal RNA gene	Deltaproteobacteria bacterium	444	444	100%	3.E-120	99.2	1374	OQ808150.1	tidal flat sediment, South Korea Ganghwa island	
	Deltaproteobacteria bacterium strain IMCC39484 16S ribosomal RNA gene	Deltaproteobacteria bacterium	435	435	100%	2.E-117	98.41	1428	OQ808171.1	tidal flat sediment, South Korea Ganghwa island	
	Delta Proteobacterium G50VI partial 16S rRNA gene	delta proteobacterium G50VI	417	417	100%	5.E-112	96.81	705	AJ786070.1	subsurface sediment tidal flat; North Sea	(Köpke et al., 2005)
	Desulfuromonas acetoxidans strain DSM 684 16S ribosomal RNA	Desulfuromonas acetoxidans	413	413	100%	2.E-110	96.41	1558	NR_121678.1	Antarctic sediment South Orkney Islands	(Pfennig and Biebl, 1976)
	Deltaproteobacteria bacterium strain IMCC39542 16S ribosomal RNA gene	Deltaproteobacteria bacterium	404	404	100%	1.E-107	95.62	1372	OQ808218.1	tidal flat sediment, South Korea Ganghwa island	
	Deltaproteobacteria bacterium strain IMCC39489 16S ribosomal RNA gene	Deltaproteobacteria bacterium	404	404	100%	1.E-107	95.62	1478	OQ808176.1	tidal flat sediment, South Korea Ganghwa island	
	Pelobacter sp. 16S rRNA gene	Pelobacter sp. A3b3	404	404	100%	1.E-107	95.62	1551	AJ271656.1	Black Sea shelf sediments	(Thamdrup et al., 2000)
	Delta proteobacterium S1 16S ribosomal RNA gene	Desulfuromusa sp. S1	368	368	100%	8.E-97	92.43	1436	AY187309.1	current-harvesting electrode in marine fuel cell	(Holmes et al., 2004)

Table S6: Sequence similarities of *Desulfuromusa* ASV from this study compared to *Desulfuromusa* type strains and ASVs and OTUs from other studies from Potter Cove. In the bottom left corner percent identity is displayed, in the top right corner the coverage in percent of the query to the sequence. The ASV sq30 from this study is outlined by thicker border lines. See text section 1.3.1 for ASV sequences of Potter Cove *in situ* (PC in situ) and OTU sequences of the SIP experiment with Potter Cove sediment (PC macroalgae (Aromokeye et al., 2021)). Accession numbers for used type strain sequences: *D.bakii* ENA X79412.1; *D.kysingii* ENA X79414.1; *D.succinoxidans* ENA X79415.1; *D.ferrireducens* ENA AY835392.1

percent identity\coverage	**D.bakii**	**D.kysingii**	**D.succinoxidans**	**D.ferrireducens**	**Mn inc sq30**	**PC in situ sq402**	**PC in situ sq875**	**PC macroalgae OTU33687944532408**	**PC macroalgae OTU76210975478505**
**D.bakii**	/	100	100	100	100	100	100	100	100
**D.kysingii**	98.89	/	100	100	100	100	100	100	100
**D.succinoxidans**	98.19	98.19	/	100	100	100	100	100	100
**D.ferrireducens**	95.45	95.55	95.9	/	100	100	100	100	100
**Mn inc sq30**	99.6	98.01	98.01	97.21	/	100	100	93	90
**PC in situ sq402**	98.01	98.41	99.2	98.41	98.01	/	100	93	90
**PC in situ sq875**	99.6	98.01	98.01	97.21	100	98.01	/	93	90
**PC macroalgae OTU33687944532408**	99.58	97.92	97.92	97.08	100	97.78	100	/	80
**PC macroalgae OTU76210975478505**	98.75	97.08	97.08	96.25	100	97.71	100	100	/

3 References

Altschul, S.F., Madden, T.L., Schäffer, A.A., Zhang, J., Zhang, Z., Miller, W., et al. (1997). Gapped BLAST and PSI-BLAST: a new generation of protein database search programs. Nucleic Acids Res. 25:17**,** 3389-3402. doi: 10.1093/nar/25.17.3389

Aromokeye, D.A., Willis-Poratti, G., Wunder, L.C., Yin, X., Wendt, J., Richter-Heitmann, T., et al. (2021). Macroalgae degradation promotes microbial iron reduction via electron shuttling in coastal Antarctic sediments. Environ. Int. 156**,** 106602. doi: 10.1016/j.envint.2021.106602

Baek, K., Jang, S., Chung, E.J., Ryu, S.H., and Choi, A. (2023). Poseidonibacter ostreae sp. nov., Isolated from the Gut of Ostrea from the Seomjin River. Diversity 15:8**,** 920.

Canfield, D.E., Thamdrup, B., and Hansen, J.W. (1993). The anaerobic degradation of organic matter in Danish coastal sediments: iron reduction, manganese reduction, and sulfate reduction. Geochim. Cosmochim. Acta 57:16**,** 3867-3883. doi: 10.1016/0016-7037(93)90340-3

Cho, H., Kim, B., Mok, J.S., Choi, A., Thamdrup, B., and Hyun, J.H. (2020). Acetate-utilizing microbial communities revealed by stable-isotope probing in sediment underlying the upwelling system of the Ulleung Basin, East Sea. Mar. Ecol. Prog. Ser. 634**,** 45-61. doi: 10.3354/meps13182

Dehning, I., and Schink, B. (1989). Malonomonas rubra gen. nov. sp. nov., a microaerotolerant anaerobic bacterium growing by decarboxylation of malonate. Arch. Microbiol. 151:5**,** 427-433. doi: 10.1007/BF00416602

Gerrish, L. Data from: Automatically extracted rock outcrop dataset for Antarctica (7.3). UK Polar Data Centre, Natural Environment Research Council, UK Research & Innovation. (2020) doi: 10.5285/178ec50d-1ffb-42a4-a4a3-1145419da2bb

Guo, X.-H., Wang, N., Yuan, X.-X., Zhang, X.-Y., Chen, X.-L., Zhang, Y.-Z., et al. (2019). Poseidonibacter antarcticus sp. nov., isolated from Antarctic intertidal sediment. Int. J. Syst. Evol. Microbiol. 69:9**,** 2717-2722. doi: 10.1099/ijsem.0.003539

Han, Y., and Perner, M. (2015). The globally widespread genus Sulfurimonas: versatile energy metabolisms and adaptations to redox clines. Front. Microbiol. 6. doi: 10.3389/fmicb.2015.00989

Harrison, J.P., Schratzberger, M., Sapp, M., and Osborn, A.M. (2014). Rapid bacterial colonization of low-density polyethylene microplastics in coastal sediment microcosms. BMC Microbiol. 14:1**,** 232. doi: 10.1186/s12866-014-0232-4

Holmes, D., Bond, D., O’neil, R., Reimers, C., Tender, L., and Lovley, D. (2004). Microbial communities associated with electrodes harvesting electricity from a variety of aquatic sediments. Microb. Ecol. 48:2**,** 178-190. doi: 10.1007/s00248-003-0004-4

Jerosch, K., Scharf, F.K., Deregibus, D., Campana, G.L., Zacher-Aued, K., Pehlke, H., et al. Data from: High resolution bathymetric compilation for Potter Cove, WAP, Antarctica, with links to data in ArcGIS format. PANGAEA. (2015) doi: 10.1594/PANGAEA.853593

Jørgensen, B.B. (1977). The sulfur cycle of a coastal marine sediment (Limfjorden, Denmark). Limnol. Oceanogr. 22:5**,** 814-832. doi: 10.4319/lo.1977.22.5.0814

Jørgensen, B.B., Findlay, A.J., and Pellerin, A. (2019). The biogeochemical sulfur cycle of marine sediments. Front. Microbiol. 10:849**,** 849. doi: 10.3389/fmicb.2019.00849

Jurado, V., D'Angeli, I., Martin-Pozas, T., Cappelletti, M., Ghezzi, D., Gonzalez-Pimentel, J.L., et al. (2021). Dominance of Arcobacter in the white filaments from the thermal sulfidic spring of Fetida Cave (Apulia, southern Italy). Sci. Total Environ. 800**,** 149465. doi: 10.1016/j.scitotenv.2021.149465

Köpke, B., Wilms, R., Engelen, B., Cypionka, H., and Sass, H. (2005). Microbial Diversity in Coastal Subsurface Sediments: a Cultivation Approach Using Various Electron Acceptors and Substrate Gradients. Appl. Environ. Microbiol. 71:12**,** 7819-7830. doi: 10.1128/AEM.71.12.7819-7830.2005

Liesack, W., and Finster, K. (1994). Phylogenetic analysis of five strains of gram-negative, obligately anaerobic, sulfur-reducing bacteria and description of *Desulfuromusa* gen. nov., including *Desulfuromusa kysingii* sp. nov., *Desulfuromusa bakii* sp. nov., and *Desulfuromusa succinoxidans* sp. nov. Int. J. Syst. Evol. Microbiol. 44:4**,** 753-758. doi: 10.1099/00207713-44-4-753

Lueders, T., Manefield, M., and Friedrich, M.W. (2004). Enhanced sensitivity of DNA- and rRNA-based stable isotope probing by fractionation and quantitative analysis of isopycnic centrifugation gradients. Environ. Microbiol. 6:1**,** 73-78. doi: 10.1046/j.1462-2920.2003.00536.x

McKenzie, R.M. (1971). The synthesis of birnessite, cryptomelane, and some other oxides and hydroxides of manganese. Mineral. Mag. 38:296**,** 493-502. doi: 10.1180/minmag.1971.038.296.12

Michaud, A.B., Laufer, K., Findlay, A., Pellerin, A., Antler, G., Turchyn, A.V., et al. (2020). Glacial influence on the iron and sulfur cycles in Arctic fjord sediments (Svalbard). Geochim. Cosmochim. Acta 280**,** 423-440. doi: 10.1016/j.gca.2019.12.033

Miller, W.G., Yee, E., and Bono, J.L. (2018). Complete Genome Sequence of the Arcobacter bivalviorum Type Strain LMG 26154. Microbiology Resource Announcements 7:12**,** 10.1128/mra.01076-01018. doi: 10.1128/mra.01076-18

Monien, P., Lettmann, K.A., Monien, D., Asendorf, S., Wölfl, A.-C., Lim, C.H., et al. (2014a). Redox conditions and trace metal cycling in coastal sediments from the maritime Antarctic. Geochim. Cosmochim. Acta 141**,** 26-44. doi: 10.1016/j.gca.2014.06.003

Monien, P., Schnetger, B., and Brumsack, H.-J. Data from: Geochemistry of sediment core PC-P01b, Potter Cove, King George Island. PANGAEA. (2014b) doi: 10.1594/PANGAEA.805935

Neder, C., Fofonova, V., Androsov, A., Kuznetsov, I., Abele, D., Falk, U., et al. (2022). Modelling suspended particulate matter dynamics at an Antarctic fjord impacted by glacier melt. J. Mar. Syst. 231**,** 103734. doi: 10.1016/j.jmarsys.2022.103734

Pfennig, N., and Biebl, H. (1976). *Desulfuromonas acetoxidans* gen. nov. and sp. nov., a new anaerobic, sulfur-reducing, acetate-oxidizing bacterium. Arch. Microbiol. 110:1**,** 3-12. doi: 10.1007/BF00416962

Pjevac, P., Kamyshny Jr, A., Dyksma, S., and Mußmann, M. (2014). Microbial consumption of zero-valence sulfur in marine benthic habitats. Environ. Microbiol. 16:11**,** 3416-3430. doi: 10.1111/1462-2920.12410

Takai, K., Nunoura, T., Horikoshi, K., Shibuya, T., Nakamura, K., Suzuki, Y., et al. (2009). Variability in Microbial Communities in Black Smoker Chimneys at the NW Caldera Vent Field, Brothers Volcano, Kermadec Arc. Geomicrobiol. J. 26:8**,** 552-569. doi: 10.1080/01490450903304949

Thamdrup, B., Rosselló-Mora, R., and Amann, R. (2000). Microbial manganese and sulfate reduction in Black Sea shelf sediments. Appl. Environ. Microbiol. 66:7**,** 2888-2897. doi: 10.1128/aem.66.7.2888-2897.2000

Vandieken, V., Mußmann, M., Niemann, H., and Jørgensen, B.B. (2006). *Desulfuromonas svalbardensis* sp. nov. and *Desulfuromusa ferrireducens* sp. nov., psychrophilic, Fe(III)-reducing bacteria isolated from Arctic sediments, Svalbard. Int. J. Syst. Evol. Microbiol. 56:5**,** 1133-1139. doi: 10.1099/ijs.0.63639-0

Zhang, Z., Schartz, S., Wagner, L., and Miller, W. (2000). A Greedy Algorithm for Aligning DNA Sequences. J. Comput. Biol. 7:1-2**,** 203-214. doi: 10.1089/10665270050081478
